# Site-Selective Polyolefin Hydrogenolysis on Atomic Ru for Methanation Suppression and Liquid Fuel Production

**DOI:** 10.34133/research.0032

**Published:** 2023-01-13

**Authors:** Mingyu Chu, Xianpeng Wang, Xuchun Wang, Xiangxi Lou, Congyang Zhang, Muhan Cao, Lu Wang, Youyong Li, Sibao Liu, Tsun-Kong Sham, Qiao Zhang, Jinxing Chen

**Affiliations:** ^1^ Institute of Functional Nano & Soft Materials (FUNSOM), Jiangsu Key Laboratory of Advanced Negative Carbon Technologies, Joint International Research Laboratory of Carbon-Based Functional Materials and Devices, Soochow University, Suzhou 215123, P. R. China.; ^2^ Department of Chemistry, University of Western Ontario, London, Ontario N6A 5B7, Canada.; ^3^ Key Laboratory of Superlight Materials and Surface Technology, Ministry of Education, College of Material Science and Chemical Engineering, Harbin Engineering University, Harbin 150001, China.; ^4^ Key Laboratory for Green Chemical Technology of Ministry of Education, School of Chemical Engineering and Technology, Tianjin University, Tianjin 300072, China.

## Abstract

Catalytic hydrogenolysis of end-of-life polyolefins can produce value-added liquid fuels and therefore holds great promises in plastic waste reuse and environmental remediation. The major challenge limiting the recycling economic benefit is the severe methanation (usually >20%) induced by terminal C–C cleavage and fragmentation in polyolefin chains. Here, we overcome this challenge by demonstrating that Ru single-atom catalyst can effectively suppress methanation by inhibiting terminal C–C cleavage and preventing chain fragmentation that typically occurs on multi-Ru sites. The Ru single-atom catalyst supported on CeO_2_ shows an ultralow CH_4_ yield of 2.2% and a liquid fuel yield of over 94.5% with a production rate of 314.93 g_fuels_ g_Ru_^−1^ h^−1^ at 250 °C for 6 h. Such remarkable catalytic activity and selectivity of Ru single-atom catalyst in polyolefin hydrogenolysis offer immense opportunities for plastic upcycling.

## Introduction

The ever-growing plastic demands of modern society cause the production of massive solid waste, threatening the environment on which we depend [1–4]. Polyolefins, used mainly in containers, tubes, and packaging films, account for 57% of the total plastic commodity market [5–7]. However, over 85% of waste polyolefins are dumped in landfills or disposed of in environmentally harmful ways (e.g., incineration), contradicting the global sustainability efforts since polyolefin is another form of fossil energy [8–10]. The catalytic hydrogenolysis reaction has been developed to convert polyolefins into value-added liquid fuels under mild conditions efficiently [11–16]. The H_2_-rich system promotes the hydrogenation of cracked intermediate to form alkanes and suppresses the coke deposition [17]. Up to now, ruthenium (Ru) catalysts manifest the highest catalytic activity for polyolefin hydrogenolysis because they can simultaneously activate all reaction steps, including (a) C−H dehydrogenation, (b) C–C cleavage, and (c) hydrogenation [18–22]. Nevertheless, severe methanation of polyolefins (usually >20%) on multisite Ru has remained a significant challenge to the industrialization of the recycling technology, as it limits the production of higher-value liquid fuels [11,12,23]. While lowering the reaction temperature and increasing H_2_ pressure can kinetically alleviate methanation, it is impractical because of the much longer reaction time and higher equipment requirements [24,25].

Recently, geometric control of active metal has been reported to significantly tune the catalytic performance, including activity, selectivity, and stability [26,27]. Smaller metal size and higher disordered properties can boost the production of liquid fuels and suppress the methanation [14,28]. In general, methanation is mainly caused by the dissociation of the terminal C_secondary_–C_primary_ bond and the fragmentation of the chain on conventional multisite Ru catalysts [25,29–31]. First, the continuous cleavage of terminal carbon from the polymer chain generates methane [14]. Although the limited chain ends in the initial stage do not produce much methane, as the reaction proceeds, a surge in the number of chain ends leads to substantial methane production. Second, simultaneous cracking of 2 adjacent C–C bonds can be catalyzed by clustered Ru atoms. This process is unavoidable on the multisite Ru catalyst surface. Therefore, eliminating the adsorption of polyolefin chains on multisite Ru catalysts or decreasing the energy barrier of internal chain cleavage is expected to suppress the methanation reaction in polyolefin hydrogenolysis.

We show here that the rational design of Ru single-atom catalyst (Ru SAC) provides a solution to the challenge because of its isolated Ru structure and site-selective cleavage mode. Mechanism studies reveal that the internal C–C cleavage is more energy favorable on Ru SAC, thus suppressing the terminal cracking to produce methane; the Ru single-atom cannot provide enough coordination space, thus preventing the continuous breaking of adjacent C–C bonds and inhibiting the production of methane. As a result, the Ru SAC exhibited an ultralow CH_4_ yield of 2.2% and the liquid fuel yield of 94.5%. On the basis of this structure–performance relationship, the single-atom system is also universal for efficient upcycling of commercial polyolefins to liquid fuels, offering a promising prospect for plastic upcycling.

## Results

### Synthesis and morphological characterizations of Ru catalysts

**Fig. 1.**
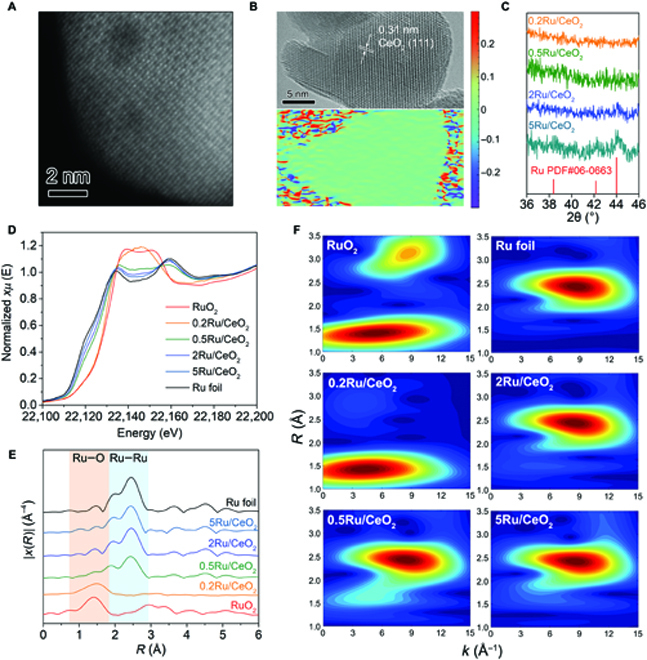
Structural characterizations of various Ru catalysts. (A) Aberration-corrected high-angle annular dark-field scanning transmission electron microscopy image of 0.2Ru/CeO_2_ catalyst and (B) high-resolution transmission electron microscopy image of CeO_2_ in 0.2Ru/CeO_2_ catalyst and corresponding strain distributions of the shear component (*ε_xy_*) determined by a geometric phase analysis. (C) High-resolution XRD patterns of Ru catalysts ranged from 36° to 46°. (D) XANES spectra, (E) EXAFS spectra, and (F) wavelet transform contour plots of various Ru catalysts and references of Ru foil and RuO_2_.

Ru/CeO_2_ catalysts with different Ru loadings (0.2, 0.5, 2, and 5 wt%, named as 0.2Ru/CeO_2_, 0.5Ru/CeO_2_, 2Ru/CeO_2_, and 5Ru/CeO_2_, respectively) were synthesized by wet-impregnation method. The RuCl_3_ aqueous solution was dropped onto the commercial CeO_2_ nanoparticles with an average diameter of ~30 nm, followed by dry, calcination, and reduction steps. Ru contents were determined by inductively coupled plasma optical emission spectrometry with similar values to feed ratios (Table S1). The morphological information of 0.2Ru/CeO_2_ was first collected by aberration-corrected high-angle annular dark-field scanning transmission electron microscopy (Fig. 1A). While subtle differences in contrast between Ru and CeO_2_ make the observation difficult, no identifiable Ru cluster and nanoparticle indicate its high dispersity, which is consistent with the results of energy-dispersive x-ray spectroscopy (Fig. S1). Interestingly, the other 3 catalysts with much higher Ru ratios share a similar phenomenon: Ru species distribute uniformly on the CeO_2_ supports (Fig. S2). The strong metal–support interaction (SMSI) between Ru and CeO_2_ should contribute to dispersing Ru atoms [32,33], and such an SMSI effect would also enhance the catalytic stability (see detailed discussion in catalysis part).

The crystal structure of support was also visualized by high-resolution transmission electron microscopy (Fig. 1B). The clear lattice fringe with a distance of 0.31 nm is indexed to the (111) facet of CeO_2_. To quantify strain fields in crystalline lattices at a nanoscale resolution, we employed geometric phase analysis (see part 1.3 in the Supplementary Materials). The strain distributions on the single nanoparticle indicate no defect on support after calcination and reduction processes, thus ruling out the substrate effect on the catalytic performance. The high crystallinity of support is also confirmed by the sharp and strong peaks of CeO_2_ in the x-ray diffraction (XRD) patterns (PDF no. 34-0394; Fig. S3). The high-resolution XRD pattern of 5Ru/CeO_2_ in the range of 36° to 46° (Fig. 1C) also has several weak peaks located at 38.4°, 42.2°, and 44.0°, corresponding to the (100), (002), and (101) facets of hexagonal close packed (hcp) Ru nanocrystals (PDF no. 06-0663). It is worth noting that the signal-to-noise ratio of these diffraction peaks decreases sharply with decreased Ru loading, which suggests that the dispersibility of Ru atoms on the CeO_2_ surface increases. In particular, the 0.2Ru/CeO_2_ catalyst does not even have an identifiable diffraction signal; thus, it is expected to have a highly dispersed structure.

### Electronic structures of Ru catalysts

The electronic property of Ru species plays a significant effect on adsorption/activation/desorption behaviors of each reaction process [34,35]; it could also act as persuasive evidence to identify the single-atom structure [36,37]. Therefore, x-ray photoelectron spectroscopy was performed at Ru 3p orbitals (Fig. S4A), since the Ru 3d orbitals are partially overlapped with C 1s orbital. The fine electronic structure of Ru 3p orbitals shows that the species abundance of Ru^4+^ (463.4 eV, oxidative state) and Ru^0^ (461.8 eV, metallic state) is related to the loading amount [38]. The proportion of Ru^4+^ increases significantly with decreasing loading, while the trend of Ru^0^ species is in reverse. The decrease in the loading causes a smaller cluster size, and the aggregated Ru ensembles are broken and isolated, thus promoting the formation of the Ru–O coordination structure. In contrast, the 0.2Ru/CeO_2_ catalyst exhibits only an oxidation state (Ru^4+^) located at 463.4 eV after high-temperature reduction treatment, suggesting its isolated Ru atom structure combined with O from CeO_2_ or a subnanometer cluster structure. On the other hand, all Ru/CeO_2_ catalysts exhibit almost the same Ce 3d orbitals (Fig. S4B), and the CeO_2_ support cannot be reduced before ~350 °C measured by H_2_-temperature program reduction (Fig. S5), which suggests that there are no oxygen vacancies formation during the reduction treatment (300 °C) and hydrogenolysis process (250 °C). The above results exclude the influence of support variation on the catalytic performance.

To further study the precise local electronic structures, we collected x-ray absorption fine structure spectra, including x-ray absorption near-edge structure (XANES) and extended x-ray absorption fine structure (EXAFS), at Ru K-edge. Ru foil and RuO_2_ were used as references. As shown in Fig. 1D, with decreasing the Ru loading, the threshold energy (1s → 5p state transition) shifts to the higher energy accompanied by enhanced whiteline intensity, indicating a continuous species evolution from a metallic state to an oxidation state toward RuO_2_. The broadened post-edge feature around 22,160 eV suggests a shrinkage in the cluster size for the samples with lower Ru loading. When the Ru loading is reduced to 0.2 wt%, the blurred spectral feature compared to that of RuO_2_ implies its distinct local structure and smaller ensemble size.

Fourier transform (phase uncorrected) is performed on the EXAFS to investigate the local coordination environment of Ru species (Fig. 1E). Remarkably, only the Ru–O scattering path is observed in the 0.2Ru/CeO_2_ catalyst, providing solid evidence for the formation of Ru SAC [39,40]. By contrast, the Ru nanocluster catalysts show obvious Ru–Ru scattering paths at ~ 2.4 Å and weak Ru–O scattering paths at ~1.4 Å, confirming their clustered states. Wavelet transform, a parallel mathematical transformation to Fourier transform but with an extra dimension (i.e., energy), is performed upon the EXAFS spectra to precisely identify scattering paths (Fig. 1F) [41]. 0.2Ru/CeO_2_ shares the same intensity maximum located at *k* = 5 Å^−1^ and *R* = 1.4 Å with that of RuO_2_, which should be assigned to Ru–O scattering path. However, no features are observed at *k* = 8.8 Å^−1^ and *R* = 2.4 Å, excluding the existence of Ru–Ru scattering path and evidencing its single-atom feature again, whereas other samples with higher Ru loading show similar intensity maxima with that of Ru foil, further demonstrating their cluster intrinsicality. A quantitative analysis was carried out by fitting the EXAFS spectra in the R space (Table S2). In the case of 0.2Ru/CeO_2_ catalyst, Ru is only coordinated to O with an average coordination number of 4.59, and no other scattering path can be observed. For all nanocluster catalysts, the Ru coordination numbers are in the range of 6 to 8, which is much lower than that of the fully occupied Ru foil [coordination number (CN) = 12]. Meanwhile, its decreasing trend when reducing Ru content confirms that the Ru cluster size becomes smaller. The Ru–Ru bond distance (2.67 Å) is the same as that of Ru foil. The O coordination numbers are all lower than 2, which should be assigned to the trace amount of oxygen species sitting on the cluster surface.

### Hydrogenolysis of polyethylene on Ru catalysts

**Fig. 2.**
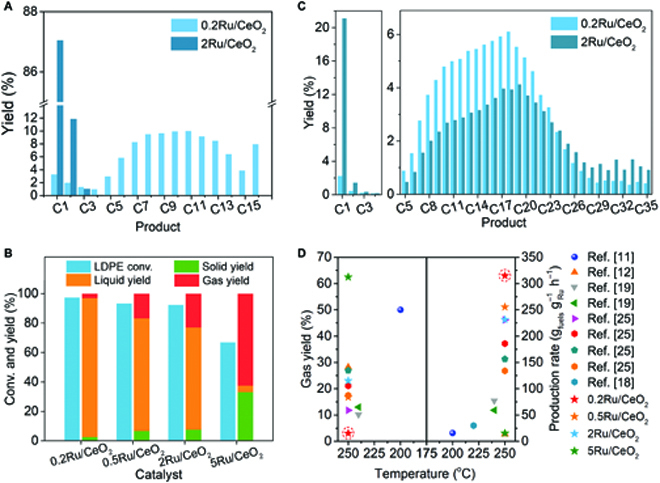
Catalytic performances of Ru catalysts for LDPE hydrogenolysis. (A) Catalytic performance of Ru SAC and 2Ru/CeO_2_ on *n*-hexadecane hydrogenolysis. Reaction conditions: *T* = 240 °C, *t* = 8 h, *P*_H2_ = 2 MPa, stirring rate = 400 rpm, *m*(Ru) = 1 mg, and *n*(*n*-hexadecane) = 10 mmol. (B) Catalytic performance of various Ru catalysts on LDPE hydrogenolysis. (C) Products yields of Ru SAC and 2Ru/CeO_2_ on LDPE hydrogenolysis. Reaction conditions: *T* = 250 °C, *t* = 6 h, *P*_H2_ = 2 MPa, stirring rate = 400 rpm, and *m*(Ru)/*m*(LDPE) = 1 mg/2,000 mg. (D) Comparison of gas yield and production rate {*m*(PE or PP) × liquid yield / [*m*(catalyst) × *t*]} of liquid fuels on Ru catalysts for polyolefin hydrogenolysis.

We first applied *n*-hexadecane as a model reactant and investigated its hydrogenolysis performance over both Ru SAC and 2Ru/CeO_2_ catalyst in a batch stainless steel autoclave (Fig. S6). The 87% of methane yield and nearly 100% of gas yield indicate that 2Ru/CeO_2_ tends to cleave the terminal carbons of *n*-hexadecane (Fig. 2A). Under the same reaction conditions, the Ru SAC provides products with a normal distribution centered on C8, dominated by the internal cracking model. Then, the catalytic hydrogenolysis of low-density polyethylene (LDPE; melting index = 20 to 30 g 10 min^–1^) over various Ru catalysts was systematically tested. The feedstock ratios of Ru to LDPE in all cases were kept at 1:2,000 mg, and the reaction temperature and the H_2_ pressure were set to 250 °C and 2 MPa. As shown in Fig. 2B, the Ru SAC exhibits the highest LDPE solid conversion and liquid yield of 97.5% and 94.5%, respectively, as well as the lowest gas yield of 3.05%. Continuous dropped activities and increased overhydrogenolysis tendency are observed with increasing Ru loading. In particular, the solid conversion and liquid product yield of the 5Ru/CeO_2_ catalyst drop to only 67.0% and 4.5%, while more than 62.5% of the products are gases. The changing trend of catalytic performance is consistent with the structural evolution from the single-atom structure to clustered Ru aggregates, which confirms that Ru SAC achieves site-selective cleavage of PE chains.

The product distributions of collected gases and liquids on Ru SAC and 2Ru/CeO_2_ catalyst with high activities were analyzed by gas chromatography. As seen by visual inspection and statistical analysis of the product distributions (Fig. 2C), the Ru SAC provided a C16-centered distribution of liquid products and the lowest methane yield of 2.2%, whereas a broad C18-centered liquid distribution and 21.1% of methane were received from 2Ru/CeO_2_. The relatively narrow product distribution and lower methane yield obtained from Ru SAC indicate the internal C–C cleavage model on the Ru single-atom site, leading to the rapid shrink of chain distribution.

We further compare the catalytic performance of our Ru catalysts with that of the reported ones. The production rate of liquid fuels (in grams of fuels per gram of Ru per hour) is defined to evaluate various Ru catalysts (Fig. 2D and Table S3). Specifically, the Ru SAC has the lowest gas yield of 3.05% and the highest liquid fuel production rate of 314.93 g_fuels_ g_Ru_^−1^ h^−1^, which is far superior to other reported Ru catalysts. The 2Ru/CeO_2_ catalyst has a similar gas yield of 23.1% to the reference nanocluster catalysts, confirming that our results are reasonable. Further increasing the Ru loading (5Ru/CeO_2_) leads to a dropping in solid conversion and more tendency to methanation (gas yield of 62.5%). It is mainly due to less exposed active sites and the formation of more Ru–Ru sites to crack the terminal C–C bonds. Therefore, benefiting from the maximum atom utilization and selective cracking model, the activity and selectivity of Ru SAC are significantly improved compared to those of nanocluster catalysts.

### Hydrogenolysis mechanism of PE on Ru SAC

**Fig. 3.**
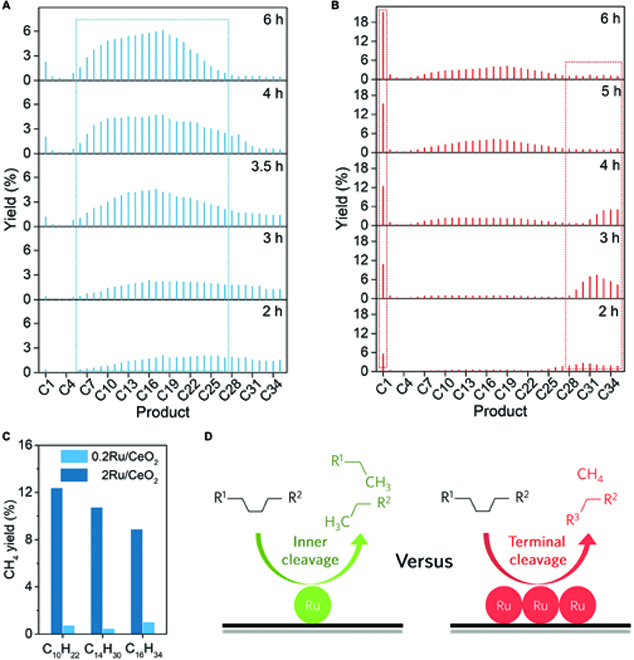
Mechanism study of Ru SAC for PE hydrogenolysis. (A and B) Distributions of nonsolid products on LDPE hydrogenolysis from 2 to 6 h over (A) Ru SAC and (B) 2Ru/CeO_2_ catalyst. Reaction conditions: *T* = 250 °C, *P*_H2_ = 2 MPa, stirring rate = 400 rpm, and *m*(Ru)/*m*(LDPE) = 1 mg/2,000 mg. (C) CH_4_ yields in hydrogenolysis of alkanes with different chain lengths on Ru SAC and 2Ru/CeO_2_. Reaction conditions: *T* = 250 °C, *P*_H2_ = 2 MPa, *t* = 3 h, stirring rate = 400 rpm, and *m*(Ru)/*m*(alkane) = 1 mg/2,000 mg. (D) Summarized cleavage modes of polyolefin on conventional SAC and nanocluster catalyst.

The in-depth mechanism investigation of the reaction pathways on Ru SAC and 2Ru/CeO_2_ is further explored by time-dependent experiments. In the initial hydrogenolysis process (less than 2 h), Ru SAC converts waste PE to products with flat and broad carbon distribution (Fig. 3A), indicating the random cleavage of the internal C–C bonds on the Ru single-atom structure. In contrast, the 2Ru/CeO_2_ nanocluster catalyst exhibits an uneven carbon distribution with both high contents of alkanes of C28 to C35 and CH_4_ (5.5%; Fig. 3B). Importantly, in the next 2 h, with a dramatic increase in solid conversion, the product distribution on the Ru SAC is shifted to the lower carbon chain with a carbon distribution center at C14, whereas the 2Ru/CeO_2_ catalyst still continuously produces C28 to C35 and CH_4_. Further extending the reaction time makes the product distribution on Ru SAC concentrate more on C14, while those C28 to C35 are digested to generate CH_4_ on 2Ru/CeO_2_ surface dramatically. The above results again confirm that the Ru SAC prefers to cleave internal C–C bonds and the terminal cleavage model on multisite Ru catalysts is dominated.

In addition, we perform the hydrogenolysis behavior on different light alkanes to further probe the mechanism. When all reaction conditions are kept the same, the CH_4_ yields in different cleavage models are sensitive to the number of terminal sites (determined by molecular weight) in the reaction system. As displayed in Fig. 3C, the CH_4_ yield on the 2Ru/CeO_2_ catalysts shows a more obvious sensitivity to the carbon-chain length that the CH_4_ yield dramatically increases from 8.9% in *n*-decane hydrogenolysis to 12.4% in *n*-hexadecane hydrogenolysis, which verifies its terminal cracking model again. Remarkably, the Ru SAC maintains stable CH_4_ yields of 0.4% to 1% during the hydrogenolysis of alkanes with different carbon numbers, confirming its inertness to terminal C–C dissociation.

To deeply explore the relationship between Ru structure and CH_4_ selectivity in the hydrogenolysis process, we performed density functional theory (DFT) calculations to understand the cleavage models of terminal/inner carbons from the polymer chain. Here, we have constructed single-atom (one Ru atom is boned with 3 surface O atoms and one O atom) and multi-Ru (Ru trimer; each Ru atom is bonded with one surface O atom) sites anchored on the CeO_2_ (111) surface to simulate the Ru SAC and Ru nanocluster catalyst. The *n*-hexane is employed to simulate the long-chain alkane for simplicity. Considering the C–H bond in *n*-hexane is easy to break on Ru atom with the calculated barriers of 0.45 eV for the terminal C1 and 0.54 eV for the inner C3 (Fig. S7), respectively. Thus, hexene is chosen as the reactants to study the cleavage mode on Ru SAC and Ru_3_/CeO_2_.

For the Ru single-atom model, when one carbon atom in hexene is bonded with a single Ru atom, Ru SAC prefers to cleave the inner C3–C4 bond with the barrier of 1.20 eV, remarkably lower than the barrier of 1.72 eV to break the terminal C1–C2 bond in hexane (Fig. S8A). A similar result is founded when Ru atom is bonded with 2 contiguous carbon atoms (Fig. S8B). Therefore, on Ru SAC, the *n*-hexane tends to dehydrogenate into 3-hexyl intermediate and cleaves the inner C–C bond in hexane, which efficiently suppresses the CH_4_ formation and facilitates the liquid fuel production. Conversely, as shown in Fig. S9A, Ru_3_/CeO_2_ prefers to cleave the terminal C1–C2 bond with the barrier of 1.54 eV rather than the inner C3–C4 bond of 1.75 eV. The *CH_2_ intermediate is formed after the C1–C2 bond breakage, implying the possible production of CH_4_. The similar results are also obtained in the cleavage of hexyne as alkyne intermediates (Fig. S9B), indicating a favorable terminal C–C cleavage mode on multi-Ru site. Combined with our experimental results, in the initial stage of alkane/polyolefin hydrogenolysis, the terminal cracking is limited by the minimal sites, and the hydrogenolysis process may undergo the random inner cracking. However, as the reaction proceeds, rapidly enhanced terminal C_secondary_–C_primary_ sites would cause serious energy-favorable methanation. The theoretical results have demonstrated that the catalytic mechanism of *n*-hexane on single-atom Ru_1_/CeO_2_ and Ru_3_/CeO_2_ surfaces is different and the single metal Ru site could inhibit the fragmentation reaction, promoting the liquid fuel production (Fig. 3D).

In addition, the whole reaction paths of *n*-hexane dissociated on the Ru SAC surface are shown in Fig. S10, and *n*-hexane first adsorbs on the Ru SAC, where the C3 atom locates on the top of the Ru atom. Under the activation of single-atom Ru, the inner C–H bond in *n*-hexane is first cleaved by overcoming a low barrier of 0.54 eV (S0 to S1), and then breaking the C3–C4 bond needs to overcome a barrier of 1.41 eV (S1 to S2) with the formation of *CH_3_CH_2_CH_2_ and *CHCH_2_CH_3_ intermediates. After that, the hydrogenation of *CH_2_CH_2_CH_3_ generates desorbed propane (S2 to S3) with a low barrier of only 0.48 eV, suggesting that the subsequent hydrogenation and desorption processes are facile once the C–C bond is activated. Our theoretical results further confirm that the internal C–C bond cleavage in *n*-hexane on Ru SAC is energetically preferred, which could successfully suppress the CH_4_ production and boost the production of liquid fuels with high selectivity.

### Controlling carbon distribution of products

**Fig. 4.**
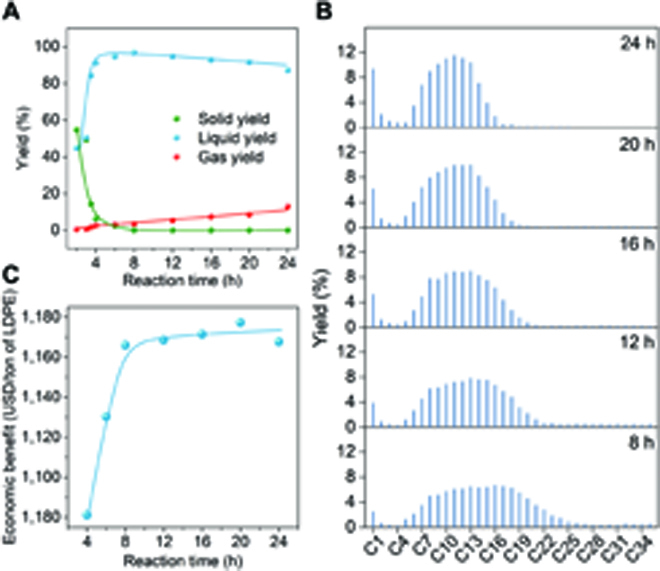
Reaction parameter optimization and products economy analysis. (A and B) Time-dependent analysis of product yields over Ru SAC on LDPE hydrogenolysis for 2 to 24 h. Reaction conditions: *T* = 250 °C, *P*_H2_ = 2 MPa, stirring rate = 400 rpm, and *m*(Ru)/*m*(LDPE) = 1 mg/2,000 mg. (C) Corresponding economic analysis based on the time-dependent experiments.

The pursuit of high recycling benefits from waste plastics and controlling product distribution are conducive to technology industrialization. Time-dependent experiments on LDPE hydrogenolysis were evaluated over Ru SAC and 2Ru/CeO_2_. After 2 h of reaction, 55% of PE has been converted to liquid alkanes (Fig. 4A). These liquid products are good solvents for PE and reduce the system viscosity, thereby enhancing mass transfer efficiency and leading to a dramatic increase in subsequent catalytic activity (during 2 to 3 h) [42,43]. The residual solids are further converted to lower alkanes in the subsequent reaction with nearly 100% of solid conversion after 6 h. Although further prolonging the reaction time increases the gas yield slightly but it remains below 10%, the large change in the carbon distribution of liquid fuel allows precise product control and value optimization. In our case, the liquid fuels ranging from C5 to C35 are divided into C5 to C12 (gasoline), C13 to C22 (diesel), and C23 to C35 (liquid wax). In general, gasoline possesses a higher value than diesel and liquid wax; thus, the hydrogenolysis process should be deepened while maintaining a low gaseous yield. As the reaction progresses to 24 h, the half-peak width of the fitted distributions becomes more concentrated, and finally, the carbon-chain center gradually shifts to C11 (Fig. 4B and Fig. S11), receiving 63% of gasoline yield (Fig. S12). Notably, while prolonging the reaction time of the 2Ru/CeO_2_ catalyst can also modulate the carbon distribution of liquid products, it leads to severe methanation, which is not conducive to the economic benefits (Fig. S13).

Corresponding economic analysis (see details in part 1.4 in the Supplementary Materials) of the products is evaluated to figure out the recycling benefits during the hydrogenolysis process. As shown in Fig. 4C, with above 90% of solid conversion of LDPE (reaction time > 4 h), the value of the overall products shows a sharp increase from 4 to 8 h, mainly due to the substantial conversion of liquid wax to the higher-value diesel and gasoline. After 8 h of reaction, the conversion of diesel to gasoline dominates the reaction. Although the values of the 2 oils are relatively close, the economic benefits only display a slow increase when prolonging the reaction time to 20 h. Noted that the subsequent overhydrogenolysis of gasoline would lead to the formation of undesired gaseous products (Fig. 4A), resulting in a decrease in product value.

While reasonable optimizations of reaction parameters are usually used to maximize the economic benefit of a specific reaction, it also affects the total energy consumption. Here, the industrial hydrogenolysis process of PE is simulated by treating 60,000 tons of PE per year (Fig. S14; see detailed flow diagram and parameters in Fig. S15 and Table S4). In a typical process, the waste PE, catalyst, and H_2_ are pumped into the reactor with 100% of solid conversion to produce the C1 to C35 hydrocarbons, followed by the separation of gases including H_2_ and C1 to C12 and the nongaseous mixture of catalyst and C13 to C35 hydrocarbons. Subsequently, the gas components pass through 2 consecutive separators to receive the H_2_, natural gas (C1 to C4), and gasoline (C5 to C12). A solid–liquid filter is used in another products line to separate the solid catalyst and the liquid C13 to C35 hydrocarbons. In addition, the liquids are transported to a rectifying tower to vaporize the diesel product (C13 to C22) with a purity of 99% and retain the liquid wax (97%). The energy consumption of each equipment and total process are calculated on the basis of the PE hydrogenolysis performance of Ru SAC for 6 h. Compared to the 3 separators and the rectifying tower, the reactor consumes the most energy which is 9.25 × 10^3^ GJ, accounting for 90.25% of the whole process (Fig. S14 and Table S5). The calculations also show that the energy consumptions for converting PE into different products (C5 to C12, C13 to C22, or C23 to C35) are dramatically different (Table S6). Although more gasoline can be obtained by prolonging the reaction time, it also leads to more energy consumption without substantially increasing the product’s value (Fig. S12). Therefore, simply prolonging the reaction time to increase gasoline yield is not conducive to increasing profits but will consume a lot of energy. The development of efficient selective catalysts can fundamentally yield the target product.

**Fig. 5.**
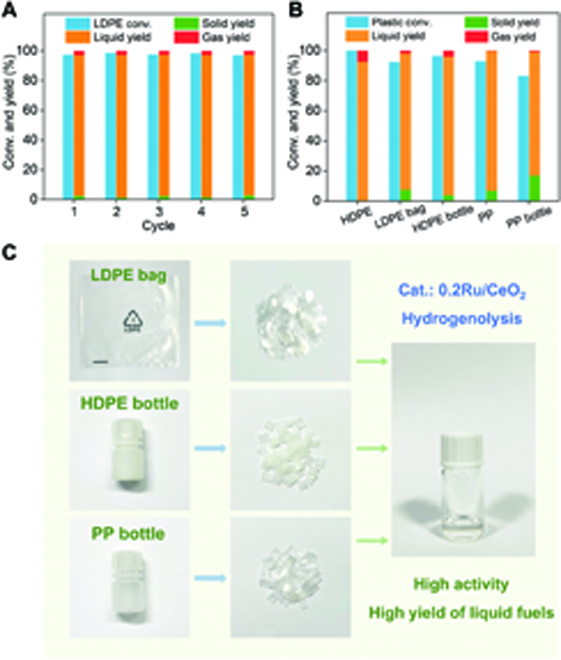
Stability and universality. (A) Stability performance of Ru SAC for LDPE hydrogenolysis. Reaction conditions: *T* = 250 °C, *P*_H2_ = 2 MPa, *t* = 6 h, stirring rate = 400 rpm, and *m*(Ru)/*m*(LDPE) = 1 mg/2,000 mg. (B) Catalytic performance and (C) schematic diagram of hydrogenolysis of various polyolefins on Ru SAC. Reaction conditions: *T* = 250 °C, *P*_H2_ = 2 MPa, stirring rate = 400 rpm, *m*(Ru)/*m*(polyolefin) = 1 mg/2,000 mg, and reaction times for HDPE, LDPE bag, HDPE bottle, PP, and PP bottle are 6, 8, 8, 18, and 18 h, respectively.

Catalyst stability and recoverability are also important criteria for industrial polyolefin hydrogenolysis. The catalytic stability of the Ru SAC was also tested. In detail, the spent Ru SAC (named Ru SAC-S) were centrifugally collected and thoroughly washed 5 times with hot toluene. The Ru SAC-S with ~10% of weight loss was then mixed with fresh quantitative catalysts for the next round of hydrogenolysis test. Over 5 hydrogenolysis cycles, Ru SAC-S exhibits almost constant catalytic performance with ~97% of solid conversion of LDPE, ~94% of liquid yield, and ~3% of gaseous yield, indicating its excellent stability (Fig. 5A). The chemical structures of the Ru SAC-S after 5 cycles were further characterized by XANES and EXAFS (Fig. S16). The spent catalyst shows only Ru–O coordination feature and a similar whiteline intensity with these of the fresh Ru SAC, indicating the stable single-atom structures. The SMSI between Ru and CeO_2_ can effectively suppress the metal sintering and leaching during the high-temperature reaction, resulting in an ultrastable hydrogenolysis performance of Ru SAC.

### Upcycling of commercial polyolefins

The feasibility and universality of Ru SAC on catalytic hydrogenolysis are investigated by degradation of high-density polyethylene (HDPE), polypropylene (PP), and commercial polyolefin waste (Fig. 5B and C). In terms of topological structure, the HDPE has a linear chain structure, while more short branches are derived in the LDPE. Under the same reaction condition, the solid conversion of HDPE reaches 100%, and the gas yield is 7.5%, exhibiting a slight overhydrogenolysis phenomenon, which indicates that catalytic hydrogenolysis of HDPE has a higher activity than that of LDPE. The low activity of LDPE may be caused by the steric hindrance of branches, which does not favor the chain adsorption on catalysts [19,30]. In addition, the methyl side groups make the breakage of backbone C–C bonds more difficult [19,44], thus requiring a longer catalytic reaction time (18 h) to achieve high solid conversion. Interestingly, the gas yield is almost zero compared to the common ~30% [12,45]. According to previous works, the PP contains many pendant methyl groups [46,47], and the removal of these groups would yield more methane than that in PE hydrogenolysis. Combined with the unique cleavage mechanism of Ru SAC proposed in the PE hydrogenolysis, we speculate that the hydrogenolysis of PP may also follow the internal C–C breakage model (right panel in Fig. S17).

Notably, since the polyolefin wastes are more complex than raw materials and always contain various additives and impurities, the hydrogenolysis of commercial plastics, including LDPE bags and HDPE and PP bottles, on Ru SAC was also demonstrated. The commercial materials were cut into squares shapes with dimensions of 5 × 5 mm for subsequent use. Overall, the Ru SAC shows high solid conversions and liquid yields over 80% and gas yields below 10% in the degradation of various commercial polyolefin wastes. Combined with the high selectivity, good stability/recyclability, and high atomic usage, the polyolefin hydrogenolysis system assisted by Ru SACs holds a great promise for promoting sustainable plastic upcycling.

## Discussion

Summarily, we have synthesized and confirmed that Ru SAC can selectively cleave internal C–C bonds in PE hydrogenolysis, whereas the terminal C–C cracking and fragmentation tend to occur at multi-Ru sites. As a result, the Ru SAC shows a production rate of 314.93 g_fuels_ g_Ru_^−1^ h^−1^ with an ultralow gaseous yield of 3.05% and a liquid fuel yield of 94.5%, which all are much better than those of other reported Ru catalysts. We have further demonstrated that the single-atom system is also universal and efficient for upcycling commercial polyolefin commodities, giving an enormous potential for industrialization. This work also offers an in-depth understanding of the structure–performance relationship between active sites and polyolefin hydrogenolysis. Our investigation of the structure–performance relationship only distinguishes the catalytic mechanism of SAC and multisite Ru catalyst, while a crucial goal for sustainable polyolefin upcycling is to develop an efficient catalyst, which corresponds to our discussion on reaction engineering parameters. In general, the catalytic performance of SAC can be modulated by tuning its electronic structure, coordination environment, and interaction with support. Therefore, rational design and study of SAC with further improved performance toward polyolefin upcycling are worth more attention.

## Materials and Methods

### Chemicals

Ruthenium(III) chloride trihydrate (RuCl_3_·3H_2_O, 99.9%), *n*-decane (*n*-C_10_H_22_, 99.9%), *n*-tetradecane (*n*-C_14_H_30_, 99.9%), and *n*-hexadecane (*n*-C_16_H_34_, 99.9%) were purchased from Alfa. Commercial nano cerium oxide (CeO_2_, 99.9%, ~30 nm) was provided from Aladdin. LDPE (melting index = 20 to 30 g/10min, size = 1,000 mesh), HDPE (melting index = 6 to 9 g/10min, size = 200 mesh), and PP (*M_n_ =* 4,000 ± 500) were purchased from Macklin. The commercial LDPE bags and HDPE bottles were brought from Alibaba Group and cut into about 0.5 × 0.5-cm flakes in prior to hydrogenolysis experiments.

### Synthesis of Ru/CeO_2_ catalysts

All Ru/CeO_2_ catalysts were prepared by a well-established wet-impregnation method. The mass fractions of Ru were kept at 0.2, 0.5, 2, and 5 wt%. In a typical synthetic process, certain amounts of RuCl_3_ aqueous solutions were dropped onto the CeO_2_ powder in a 20-ml bottle, which was put on a hotplate at 80 °C under magnetic stirring of 400 rpm until the slurry formation, followed by the dry and age in an oven at 80 °C for 12 h. Afterward, the obtained samples expected 0.2Ru/CeO_2_ were calcinated in a muffle furnace at 500 °C for 2 h. Prior to catalytic hydrogenolysis, all samples were reduced in a 5%H_2_/Ar (100 ml min^−1^) gas flow at 300 °C for 2 h.

### Catalytic evaluation and product determination

The hydrogenolysis experiments were performed in a 100-ml batch stainless steel autoclave with magnetic stirring equipment. The polymers/alkanes and catalysts were physically mixed and put into the reactor. Before catalytic tests, the reactor was preflushed with H_2_ under 2 MPa for 5 cycles, followed by the H_2_ pressurization at a total pressure of 2 MPa, and then sealed the reactor. The stirring rate during the reaction was kept at 400 rpm. After the reaction and temperature cooling down to room temperature, the gaseous products were collected by a gas sampling bag. The liquid products and solid residuals were respectively dissolved and dispersed in 30 ml of toluene and stored in the refrigerator, making the liquid of high molecular weight (carbon number > 35) is precipitated. Afterward, the solid residue and liquid product are then separated by a refrigerated centrifuge. The solid residuals were washed with *n*-hexane for 3 times and further dried in the oven at 80 °C for 12 h. For the product distribution, the gaseous (C1 to C4) and liquid (C5 to C35) products were quantitatively analyzed by 2 gas chromatographs with flame ionization detector using TM-Al_2_O_3_/S and HP-1 capillary columns, respectively. The catalytic performance was gravimetrically calculated by the following equations, the product yields are calculated on a carbon basis:

$$\text{Solid Conv.}\;\left(\%\right)=\frac{m\left(\text{plastic}\right)-m\left(\text{residuals}\right)-m\left(\text{catalyst}\right)}{m\left(\text{plastic}\right)}\times 100\%$$

$$\text{Product yield}\;\left(\%\right)=\frac{n\left(C\;\text{in}\;C_{x}\;\text{product}\right)}{n\left(C\;\text{in initial polyolefin}\right)}\times 100\%$$

### Reusability test

The solid residuals were dispersed in a hot toluene solution in a 20-ml bottle on a hotplate to dissolve the hydrocarbons. The spent catalyst was separated by centrifugation. Repeat the process 5 times to obtain the clean spent catalyst. In general, the loss of the catalyst each time was ~10%. For the new hydrogenolysis test, the catalyst was composed of all spent catalyst and partial fresh catalyst.

## Data Availability

All data needed to evaluate the conclusions in the paper are present in the paper and/or the Supplementary Materials. Additional data related to this paper may be requested from the authors.

## Supplementary Materials

Supplementary Materials Experimental Procedures. Fig. S1. Structural analysis of 0.2Ru/CeO_2_. Fig. S2. Structural analysis of 0.5Ru/CeO_2_, 2Ru/CeO_2_ and 5Ru/CeO_2_ catalysts. Fig. S3. XRD patterns of various Ru catalysts. Fig. S4. X-ray photoelectron spectroscopy spectra of various Ru catalysts at (A) Ru 3p and (B) Ce 3d orbitals. Fig. S5. H_2_-temperature program reduction curve of CeO_2_ support. Fig. S6. Picture of batch stainless steel autoclave. Fig. S7. DFT calculations on C–H activation. Fig. S8. Theoretical calculations of C–C cleavage on Ru SAC. Fig. S9. Theoretical calculations of C–C cleavage on Ru_3_/CeO_2_. Fig. S10. Catalytic reaction path and barriers of *n*-hexane dissociation on Ru SAC from DFT calculations. Fig. S11. Distribution of nonsolid products on LDPE hydrogenolysis over Ru SAC from 2 to 24 h. Fig. S12. Time-dependent analysis. Product yields of gasoline (C5 to C12), diesel (C13 to C22), and liquid wax (C23 to C35) over Ru SAC on LDPE hydrogenolysis for 4 to 24 h. Fig. S13. Time-dependent analysis of Ru non-SAC for 2 to 6 h. Fig. S14. Schematic illustration of the industrial process of PE hydrogenolysis by Aspen Plus software. Fig. S15. Detailed flow diagram of industrial PE hydrogenolysis based on the simulation through Aspen Plus software. Fig. S16. Electronic structure of Ru SAC-S. Fig. S17. Plausible mechanism of C–C hydrogenolysis of PP over Ru nanoparticle and Ru SAC. Table S1. The elemental compositions of Ru/CeO_2_ catalysts measured by inductively coupled plasma optical emission spectrometry. Table S2. Fitting results of EXAFS spectra. Table S3. Summary and compassion of various Ru-based catalysts for polyolefin hydrogenolysis. Table S4. Summary of simulation parameters. Table S5. Energy consumption distribution of each component. Table S6. Comparison of energy consumption for complete conversion of PE into different products.

## References

[1] Jia X, Qin C, Friedberger T, Guan Z, Huang Z. Efficient and selective degradation of polyethylenes into liquid fuels and waxes under mild conditions. Sci Adv. 2016;2(6):1501591. 10.1126/sciadv.1501591 PMC4928905 27386559

[2] Liu S, Kots PA, Vance BC, Danielson A, Vlachos DG. Plastic waste to fuels by hydrocracking at mild conditions. Sci Adv. 2021;7(17):eabf8283. 33883142 10.1126/sciadv.abf8283 PMC11426200

[3] García JM, Robertson ML. The future for plastic recycling. Science. 2017;358(6365):870–872. 29146798 10.1126/science.aaq0324

[4] Rahimi A, García JM. Chemical recycling of waste plastics for new materials production. Nat Rev Chem. 2017;1(6):0046.

[5] Geyer R, Jambeck JR, Law KL. Production, use, and fate of all plastics ever made. Sci Adv. 2017;3(7):1700782. 10.1126/sciadv.1700782 PMC5517107 28776036

[6] Ellis LD, Rorrer NA, Sullivan KP, Otto M, McGeehan JE, Román-Leshkov Y, Wierckx N, Beckham GT. Chemical and biological catalysis for plastics recycling and upcycling. Nat Catal. 2021;4(7):539–556.

[7] Yeung CWS, Teo JYQ, Loh XJ, Lim JYC. Polyolefins and polystyrene as chemical resources for a sustainable future: Challenges, advances, and prospects. ACS Mater Lett. 2021;3(12):1660–1676.

[8] Vollmer I, Jenks MJF, Roelands MCP, White RJ, Harmelen T, Wild P, Laan GP, Meirer F, Keurentjes JTF, Weckhuysen BM. Beyond mechanical recycling: Giving new life to plastic waste. Angew Chem Int Ed Engl. 2020;59(36):15402–15423. 32160372 10.1002/anie.201915651 PMC7497176

[9] Chu M, Liu Y, Lou X, Zhang Q, Chen J. Rational design of chemical catalysis for plastic recycling. ACS Catal. 2022;12(8):4659–4679.

[10] Duan J, Chen W, Wang C, Wang L, Liu Z, Yi X, Fang W, Wang H, Wei H, Xu S, et al. Coking-resistant polyethylene upcycling modulated by zeolite micropore diffusion. J Am Chem Soc. 2022;144(31):14269–14277. 35914188 10.1021/jacs.2c05125

[11] Rorrer JE, Beckham GT, Román-Leshkov Y. Conversion of polyolefin waste to liquid alkanes with Ru-based catalysts under mild conditions. JACS Au. 2021;1(1):8–12. 34467267 10.1021/jacsau.0c00041 PMC8395642

[12] Kots PA, Liu S, Vance BC, Wang C, Sheehan JD, Vlachos DG. Polypropylene plastic waste conversion to lubricants over Ru/TiO_2_ catalysts. ACS Catal. 2021;11(13):8104–8115.

[13] Tennakoon A, Wu X, Paterson AL, Patnaik S, Pei Y, LaPointe AM, Ammal SC, Hackler RA, Heyden A, Slowing II, et al. Catalytic upcycling of high-density polyethylene via a processive mechanism. Nat Catal. 2020;3(11):893–901.

[14] Chen L, Meyer LC, Kovarik L, Meira D, Pereira-Hernandez XI, Shi H, Khivantsev K, Gutiérrez OY, Szanyi J. Disordered, sub-nanometer Ru structures on CeO_2_ are highly efficient and selective catalysts in polymer upcycling by hydrogenolysis. ACS Catal. 2022;12(8):4618–4627.

[15] Lee W-T, van Muyden A, Bobbink FD, Mensi MD, Carullo JR, Dyson PJ. Mechanistic classification and benchmarking of polyolefin depolymerization over silica-alumina-based catalysts. Nat Commun. 2022;13(1):4850. 35977921 10.1038/s41467-022-32563-y PMC9385622

[16] Zichittella G, Ebrahim AM, Zhu J, Brenner AE, Drake G, Beckham GT, Bare SR, Rorrer JE, Román-Leshkov Y. Hydrogenolysis of polyethylene and polypropylene into propane over cobalt-based catalysts. JACS Au. 2022;2(10):2259–2268. 36311830 10.1021/jacsau.2c00402 PMC9597591

[17] Sattler JJHB, Ruiz-Martinez J, Santillan-Jimenez E, Weckhuysen BM. Catalytic dehydrogenation of light alkanes on metals and metal oxides. Chem Rev. 2014;114(20):10613–10653. 25163050 10.1021/cr5002436

[18] Jia C, Xie S, Zhang W, Intan NN, Sampath J, Pfaendtner J, Lin H. Deconstruction of high-density polyethylene into liquid hydrocarbon fuels and lubricants by hydrogenolysis over Ru catalyst. Chem Catal. 2021;1(2):437–455.

[19] Nakaji Y, Tamura M, Miyaoka S, Kumagai S, Tanji M, Nakagawa Y, Yoshioka T, Tomishige K. Low-temperature catalytic upgrading of waste polyolefinic plastics into liquid fuels and waxes. Appl Catal B Environ. 2021;285(15):119805.

[20] Wang C, Yu K, Sheludko B, Xie T, Kots PA, Vance B, Kumar P, Stach EA, Zheng W, Vlachos DG. A general strategy and a consolidated mechanism for low-methane hydrogenolysis of polyethylene over ruthenium. Appl Catal B Environ. 2022;319(15):121899.

[21] Tamura M, Miyaoka S, Nakaji Y, Tanji M, Kumagai S, Nakagawa Y, Yoshioka T, Tomishige K. Structure-activity relationship in hydrogenolysis of polyolefins over Ru/support catalysts. Appl Catal B Environ. 2022;318(5):121870.

[22] Kots PA, Xie T, Vance BC, Quinn CM, de Mello DM, Boscoboinik JA, Wang C, Kumar P, Stach EA, et al. Electronic modulation of metal-support interactions improves polypropylene hydrogenolysis over ruthenium catalysts. Nat Commun. 2022;13(1):5186. 36057603 10.1038/s41467-022-32934-5 PMC9440920

[23] Lee W-T, Bobbink FD, van Muyden AP, Lin K-H, Corminboeuf C, Zamani RR, Dyson PJ. Catalytic hydrocracking of synthetic polymers into grid-compatible gas streams. Cell Rep Phys Sci. 2021;2(2):100332.

[24] Chen L, Zhu Y, Meyer LC, Hale LV, Le TT, Karkamkar A, Lercher JA, Gutiérrez OY, Szanyi J. Effect of reaction conditions on the hydrogenolysis of polypropylene and polyethylene into gas and liquid alkanes. React Chem Eng. 2022;7(4):844–854.

[25] Wang C, Xie T, Kots PA, Vance BC, Yu K, Kumar P, Fu J, Liu S, Tsilomelekis G, Stach EA, et al. Polyethylene hydrogenolysis at mild conditions over ruthenium on tungstated zirconia. JACS Au. 2021;1(9):1422–1434. 34604852 10.1021/jacsau.1c00200 PMC8479762

[26] Wu X, Tennakoon A, Yappert R, Esveld M, Ferrandon MS, Hackler RA, LaPointe AM, Heyden A, Delferro M, Peters B, et al. Size-controlled nanoparticles embedded in a mesoporous architecture leading to efficient and selective hydrogenolysis of polyolefins. J Am Chem Soc. 2022;144(12):5323–5334. 35195400 10.1021/jacs.1c11694

[27] Le Valant A, Drault F, Maleix C, Comminges C, Beauchet R, Batonneau Y, Pirault-Roy L, Especel C, Epron F. Effect of the metallic particle size of supported Pt catalysts on methylcyclopentane hydrogenolysis: Understanding of the ring opening products distribution by a geometric approach. J Catal. 2018;367:234–243.

[28] Zhang X, Lu Y, Kovarik L, Dasari P, Nagaki D, Karim AM. Structure sensitivity of *n*-butane hydrogenolysis on supported Ir catalysts. J Catal. 2021;394:376–386.

[29] Nakagawa Y, Oya SI, Kanno D, Nakaji Y, Tamura M, Tomishige K. Regioselectivity and reaction mechanism of Ru-catalyzed hydrogenolysis of squalane and model alkanes. ChemSusChem. 2017;10(1):189–198. 27863013 10.1002/cssc.201601204

[30] Hibbitts DD, Flaherty DW, Iglesia E. Role of branching on the rate and mechanism of C–C cleavage in alkanes on metal surfaces. ACS Catal. 2016;6(1):469–482.

[31] Hibbitts DD, Flaherty DW, Iglesia E. Effects of chain length on the mechanism and rates of metal-catalyzed hydrogenolysis of *n*-alkanes. J Phys Chem C. 2016;120(15):8125–8138.

[32] Guo Y, Mei S, Yuan K, Wang D-J, Liu HC, Yan CH, Zhang YW. Low-temperature CO_2_ methanation over CeO_2_-supported Ru single atoms, nanoclusters, and nanoparticles competitively tuned by strong metal-support interactions and H-spillover effect. ACS Catal. 2018;8(7):6203–6215.

[33] Yanatake S, Nakaji Y, Betchaku M, Nakagawa Y, Tamura M, Tomishige K. Selective C-C hydrogenolysis of alkylbenzenes to methylbenzenes with suppression of ring hydrogenation. ChemCatChem. 2018;10(18):4172–4181.

[34] Yang J, Li W, Wang D, Li Y. Electronic metal-support interaction of single-atom catalysts and applications in electrocatalysis. Adv Mater. 2020;32(49):2003300. 10.1002/adma.202003300 33125802

[35] Yuan K, Guo Y, Huang L, Zhou L, Yin HJ, Liu H, Yan CH, Zhang YW. Tunable electronic metal-support interactions on ceria-supported noble-metal nanocatalysts in controlling the low-temperature CO oxidation activity. Inorg Chem. 2021;60(7):4207–4217. 33373226 10.1021/acs.inorgchem.0c03219

[36] Li X, Surkus AE, Rabeah J, Anwar M, Dastigir S, Junge H, Brückner A, Beller M. Cobalt single-atom catalysts with high stability for selective dehydrogenation of formic acid. Angew Chem Int Ed. 2020;59(37):15849–15854. 10.1002/anie.202004125 PMC7540455 32458555

[37] Kottwitz M, Li Y, Wang H, Frenkel AI, Nuzzo RG. Single atom catalysts: A review of characterization methods. Chemistry–Methods. 2021;1(6):278–294.

[38] Yao Q, Huang BL, Xu Y, Li L, Shao Q, Huang X. A chemical etching strategy to improve and stabilize RuO_2_-based nanoassemblies for acidic oxygen evolution. Nano Energy. 2021;84:105909.

[39] Lang R, Du X, Huang Y, Jiang X, Zhang Q, Guo Y, Liu K, Qiao B, Wang A, Zhang T. Single-atom catalysts based on the metal-oxide interaction. Chem Rev. 2020;120(21):11986–12043. 33112599 10.1021/acs.chemrev.0c00797

[40] Liu J. Catalysis by supported single metal atoms. ACS Catal. 2016;7(1):34–59.

[41] Muñoz M, Argoul P, Farges F. Continuous Cauchy wavelet transform analyses of EXAFS spectra: A qualitative approach. Am Mineral. 2003;88(4):694–700.

[42] Vicente G, Aguado J, Serrano DP, Sánchez N. HDPE chemical recycling promoted by phenol solvent. J Anal Appl Pyrolysis. 2009;85(1–2):366–371.

[43] Shuai L, Luterbacher J. Organic solvent effects in biomass conversion reactions. ChemSusChem. 2016;9(2):133–155. 26676907 10.1002/cssc.201501148

[44] Teh JW, Rudin A, Keung JC. A review of polyethylene-polypropylene blends and their compatibilization. Adv Polym Technol. 1994;13(1):1–23.

[45] Rorrer JE, Troyano-Valls C, Beckham GT, Román-Leshkov Y. Hydrogenolysis of polypropylene and mixed polyolefin plastic waste over Ru/C to produce liquid alkanes. ACS Sustain Chem Eng. 2021;9(35):11661–11666.

[46] Gloger D, Mileva D, Albrecht A, Hubner G, Androsch R, Gahleitner M. Long-chain branched polypropylene: Effects of chain architecture, melt structure, shear modification, and solution treatment on melt relaxation dynamics. Macromolecules. 2022;55(7):2588–2608.

[47] Chu M, Tu W, Yang S, Zhang C, Li Q, Zhang Q, Chen J. Sustainable chemical upcycling of waste polyolefins by heterogeneous catalysis. SusMat. 2022;2(2):161–185.

