# Supplementary material for: Site-Selective Polyolefin Hydrogenolysis on Atomic Ru for Methanation Suppression and Liquid Fuel Production
Source: Research (Wash D C). 2023 Jan 13;6:0032. doi: 10.34133/research.0032 (PMC10076030; doi:10.34133/research.0032)
Supplement: Supplementary Materials — Experimental Procedures. Fig. S1. Structural analysis of 0.2Ru/CeO2. Fig. S2. Structural analysis of 0.5Ru/CeO2, 2Ru/CeO2 and 5Ru/CeO2 catalysts. Fig. S3. XRD patterns of various Ru catalysts. Fig. S4. X-ray photoelectron spectroscopy spectra of various Ru catalysts at (A) Ru 3p and (B) Ce 3d orbitals. Fig. S5. H2-temperature program reduction curve of CeO2 support. Fig. S6. Picture of batch stainless steel autoclave. Fig. S7. DFT calculations on C–H activation. Fig. S8. Theoretical calculations of C–C cleavage on Ru SAC. Fig. S9. Theoretical calculations of C–C cleavage on Ru3/CeO2. Fig. S10. Catalytic reaction path and barriers of n-hexane dissociation on Ru SAC from DFT calculations. Fig. S11. Distribution of nonsolid products on LDPE hydrogenolysis over Ru SAC from 2 to 24 h. Fig. S12. Time-dependent analysis. Product yields of gasoline (C5 to C12), diesel (C13 to C22), and liquid wax (C23 to C35) over Ru SAC on LDPE hydrogenolysis for 4 to 24 h. Fig. S13. Time-dependent analysis of Ru non-SAC for 2 to 6 h. Fig. S14. Schematic illustration of the industrial process of PE hydrogenolysis by Aspen Plus software. Fig. S15. Detailed flow diagram of industrial PE hydrogenolysis based on the simulation through Aspen Plus software. Fig. S16. Electronic structure of Ru SAC-S. Fig. S17. Plausible mechanism of C–C hydrogenolysis of PP over Ru nanoparticle and Ru SAC. Table S1. The elemental compositions of Ru/CeO2 catalysts measured by inductively coupled plasma optical emission spectrometry. Table S2. Fitting results of EXAFS spectra. Table S3. Summary and compassion of various Ru-based catalysts for polyolefin hydrogenolysis. Table S4. Summary of simulation parameters. Table S5. Energy consumption distribution of each component. Table S6. Comparison of energy consumption for complete conversion of PE into different products. [file research.0032.f1.docx]

Title

Site-Selective Polyolefin Hydrogenolysis on Atomic Ru for Methanation Suppression and Liquid Fuel Production

**Authors**

Mingyu Chu^1,†^, Xianpeng Wang^1,†^, Xuchun Wang^1,2,†^, Xiangxi Lou^1,3^, Congyang Zhang^1,2^, Muhan Cao^1^, Lu Wang^1,^*, Youyong Li^1^, Sibao Liu^4^, Tsun-Kong Sham^2^, Qiao Zhang^1^, Jinxing Chen^1,^*

**Affiliations**

^1^ Institute of Functional Nano & Soft Materials (FUNSOM), Jiangsu Key Laboratory of Advanced Negative Carbon Technologies, Joint International Research Laboratory of Carbon-Based Functional Materials and Devices, Soochow University, Suzhou 215123, P. R. China.

^2^ Department of Chemistry, University of Western Ontario, London, Ontario, N6A 5B7, Canada.

^3^ Key Laboratory of Superlight Materials and Surface Technology, Ministry of Education, College of Material Science and Chemical Engineering, Harbin Engineering University, Harbin 150001, China.

^4^ Key Laboratory for Green Chemical Technology of Ministry of Education, School of Chemical Engineering and Technology, Tianjin University, Tianjin 300072, China.

^*^ Correspondence should be addressed to Lu Wang; [lwang22@suda.edu.cn](mailto:lwang22@suda.edu.cn) and Jinxing Chen; chenjinxing@suda.edu.cn

^†^ These authors contributed equally to this work

Experimental Procedures

*1.1. Characterizations.* Inductivity coupled plasma optical emission spectrometry (ICP-OES, Variance, VISTA-MPX) was used to determine the Ru loading. AC-HAADF-STEM and corresponding elemental mapping images were measured by FEI Titan Themis at 200 kV. TEM, HRTEM, HAADF-STEM, and corresponding elemental mapping images were collected by field emission high-resolution transmission electron microscopy (FEI Talos F200X, Thermo Fisher) at 200 kV. X-ray diffraction (XRD) patterns were operated through an analytical Empyrean machine at 40 kV and 40 mA. X-ray photoelectron spectroscopy (XPS) measurement was performed by a KRATOS Analytical-KRATOS ACIS Ultra DLD spectrometer using monochromatic Al Kα source with 1486.6 eV. All binding energies were calibrated by C 1s peak of adventitious carbon (284.8 eV). H_2_-temperature program reduction (H_2_-TPR) was performed on an automatic chemical adsorption instrument (FINETEC/FINE-SORB-3010). In a typically measurement, 20 mg sample was put in a U-shaped tube reactor, the sample was flushed in Ar flow to remove the physically adsorbed gases, followed by a heating reduction process from room temperature to 650 ^o^C (10 ^o^C/min) in a 5 *vol.*%H_2_/Ar flow. The outlet gases were analyzed by a thermal conductivity detector (TCD). X-ray absorption spectra at Ru *K*-edge were collected by a fluorescence yield (FY) mode at 20 BM beamline of Advanced Photon Source (APS), an office of science user facility operated for the U.S. Department of Energy Office of Science by Argonne National Laboratory, United States. All photographs were collected by a Canon 80DSLR camera.

*1.2. Computational methods.* All the density functional theory (DFT) calculations were performed by the Vienna ab initio simulation package (VASP).[1] The projected augmented wave (PAW) method was used to consider the electron−ion interactions.[2] The exchange−correlation potential was described by the Perdew−Burke−Ernzerhof (PBE) with generalized gradient approximation (GGA).[3] The van der Waals (vdW) correction of D3 was employed to describe the vdW interaction.[4] The energy cutoff was chosen as 500 eV; the criteria of energy and force convergence were 10^−4^ eV and 0.03 eV Å^−1^, respectively. A vacuum space of 20 Å was adopted to avoid the interlayer interaction. Considering the strong electron correlations in CeO_2_, our calculations were performed with the DFT+U method,[5] and the U value was set to 4.5 eV for Ce atoms according to the previous literature.[6]

The 4×4 supercell of CeO_2_ (111) surface was employed as the surface model with nine atomic layers, and the bottom six atomic layers were fixed during the structural optimization. Ru_1_/CeO_2_ (111) and Ru_3_/CeO_2_ (111) were constructed to represent the models of single-atom Ru and Ru cluster anchored on the CeO_2_ (111) surface, respectively. The Ru atom prefers to occupy the hollow site on the CeO_2_ (111) surface and bonds with three surface oxygen atoms. Transition state searches were performed using the climbing image nudged elastic band (CI-NEB) method[7].

*1.3. Theory of GPA.* The essence of GPA is the comparison of the phase of a set of perfect planes (defined by a g-vector) to the planes measured from an image (defined by a mask at the g-vector). If we consider and image to be formed from a Fourier series

$$I\left( r \right)= \sum_{g} A_{g}e^{iP_{g}+2\pi i\boldsymbol{g\cdot r}}$$

Where $I$ is the image intensity, $r$ is the position in the image and $g$ are the periodicities in the image (i.e. the position in reciprocal space). $A_{g}$ and $P_{g}$ give the amplitude and phase of the periodicity given by g. Using a simple mask, it is easy to extract one (or a select few) of these Fourier components by masking the FFT. Inversing this FFT then produces the complex image. From this, the phase difference can be calculated as

$$P_{g}\left( \boldsymbol{r} \right)=Phase\left[ H_{g}^{'}\left( \boldsymbol{r} \right) \right]-2\pi\boldsymbol{g\cdot r}$$

where the first term is the phase from the masked FFT and the second term in the phase is calculated from the g-vector the FFT was masked at. At this point, the g-vector can be refined to an area of the homogeneous strain. If the g-vector is incorrect, (even if only by a little bit!) then the phase in the uniform strain region will have a gradient. By fitting this gradient, it is possible to correct the g-vector using

$$\Delta g=\frac{1}{2\pi}\nabla P_{g}$$

Each phase can be used to calculate the displacements in the direction of the lattice place. To get the full strain field, the phase needs to be calculated for two non-colinear g-vectors. The phases are related to the displacement field, u, by

$$\left( \begin{matrix} P_{g1} \\ P_{g2} \end{matrix} \right)=-2\pi(\begin{matrix} g_{1x} & g_{1y} \\ g_{2x} & g_{2y} \end{matrix})(\begin{matrix} u_{x} \\ u_{xy} \end{matrix})$$

Where $g_{1x}$ and $g_{1y}$ are the x and y components of the g-vector using to calculate the phase, $P_{g1}$. Inverting this gives the displacements in terms of the phases,

$$\left( \begin{matrix} u_{x} \\ u_{xy} \end{matrix} \right)=\frac{1}{-2\pi}(\begin{matrix} a_{1x} & a_{2x} \\ a_{1y} & a_{2y} \end{matrix})\left( \begin{matrix} P_{g1} \\ P_{g2} \end{matrix} \right)$$

where we have used

$${(\begin{matrix} g_{1x} & g_{1y} \\ g_{2x} & g_{2y} \end{matrix})}^{T}{=(\begin{matrix} a_{1x} & a_{2x} \\ a_{1y} & a_{2y} \end{matrix})}^{-1}$$

Finally, the distortion is calculated by differentiating:

$$e=\left( \begin{matrix} e_{xx} & e_{xy} \\ e_{yx} & e_{yy} \end{matrix} \right)=\left( \begin{matrix} \frac{{\partial u}_{x}}{\partial_{x}} & \frac{{\partial u}_{x}}{\partial_{y}} \\ \frac{{\partial u}_{y}}{\partial_{x}} & \frac{{\partial u}_{y}}{\partial_{y}} \end{matrix} \right)$$

From this matrix, the strain, ε, rotation, ω and dilatation, Δ, are easily calculated from

$$\varepsilon=\frac{1}{2}(e+e^{T})$$

$$\omega=\frac{1}{2}(e-e^{T})$$

$$\Delta=Trace[e]$$

Note: the above discussion is based on the Cayman theme by Jason Long (http://jjppeters.github.io/Strainpp/)

*1.4. Economic analysis.* The market prices of natural gas, gasoline, diesel and liquid wax are collected based on the quotations of the Chinese market in April, which are 780.58, 1258.09, 1138.93 and 1115.20 USD per ton, respectively. The economic benefit refers to the value of the gas and liquid products collected per 1 ton of LDPE processed by the 0.2Ru/CeO_2_ catalyst in different reaction times.


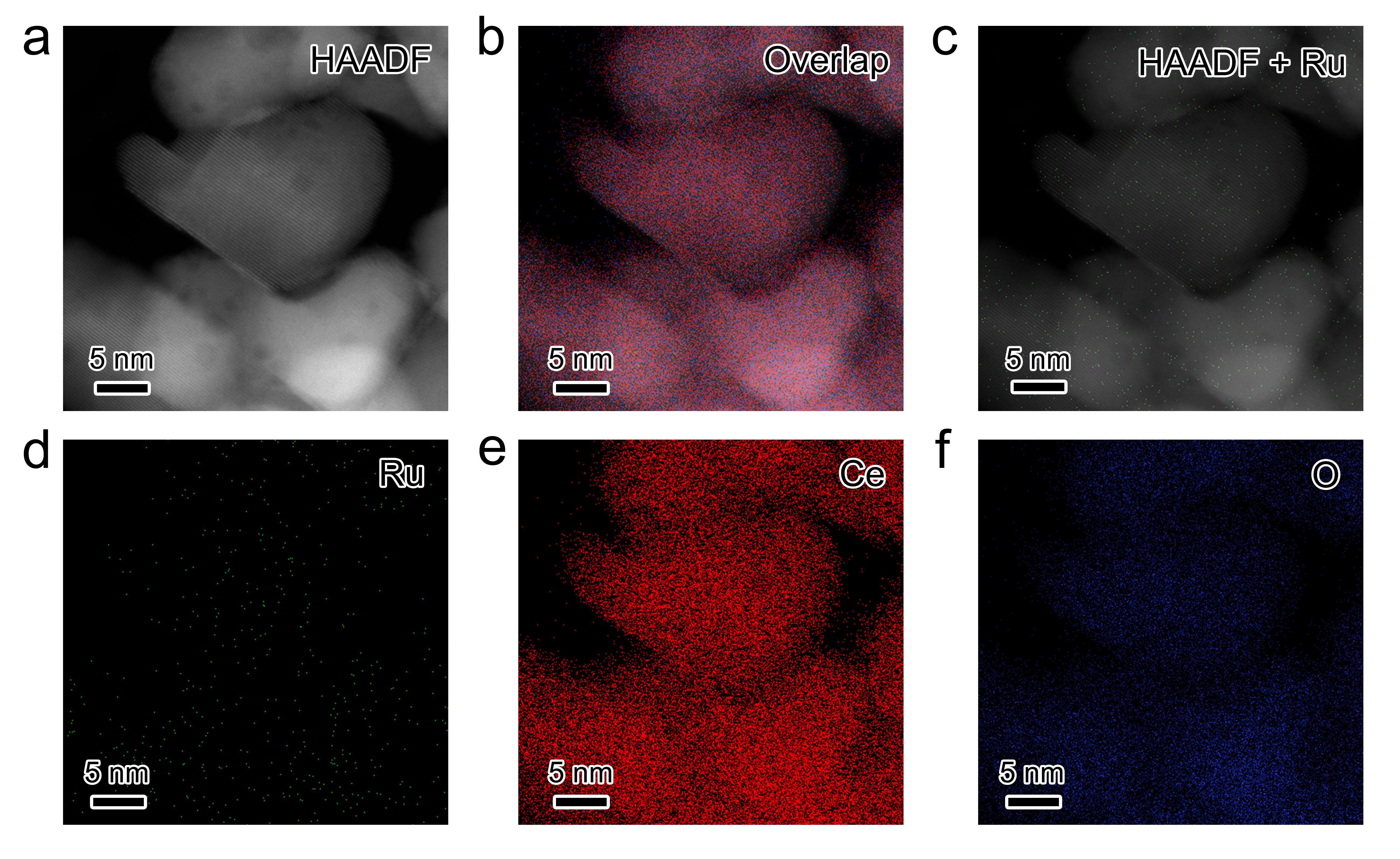


**Figure S1.** Structural analysis of 0.2Ru/CeO_2_. (a) HAADF-STEM and (b−f) elemental mapping images of 0.2Ru/CeO_2_ catalyst.


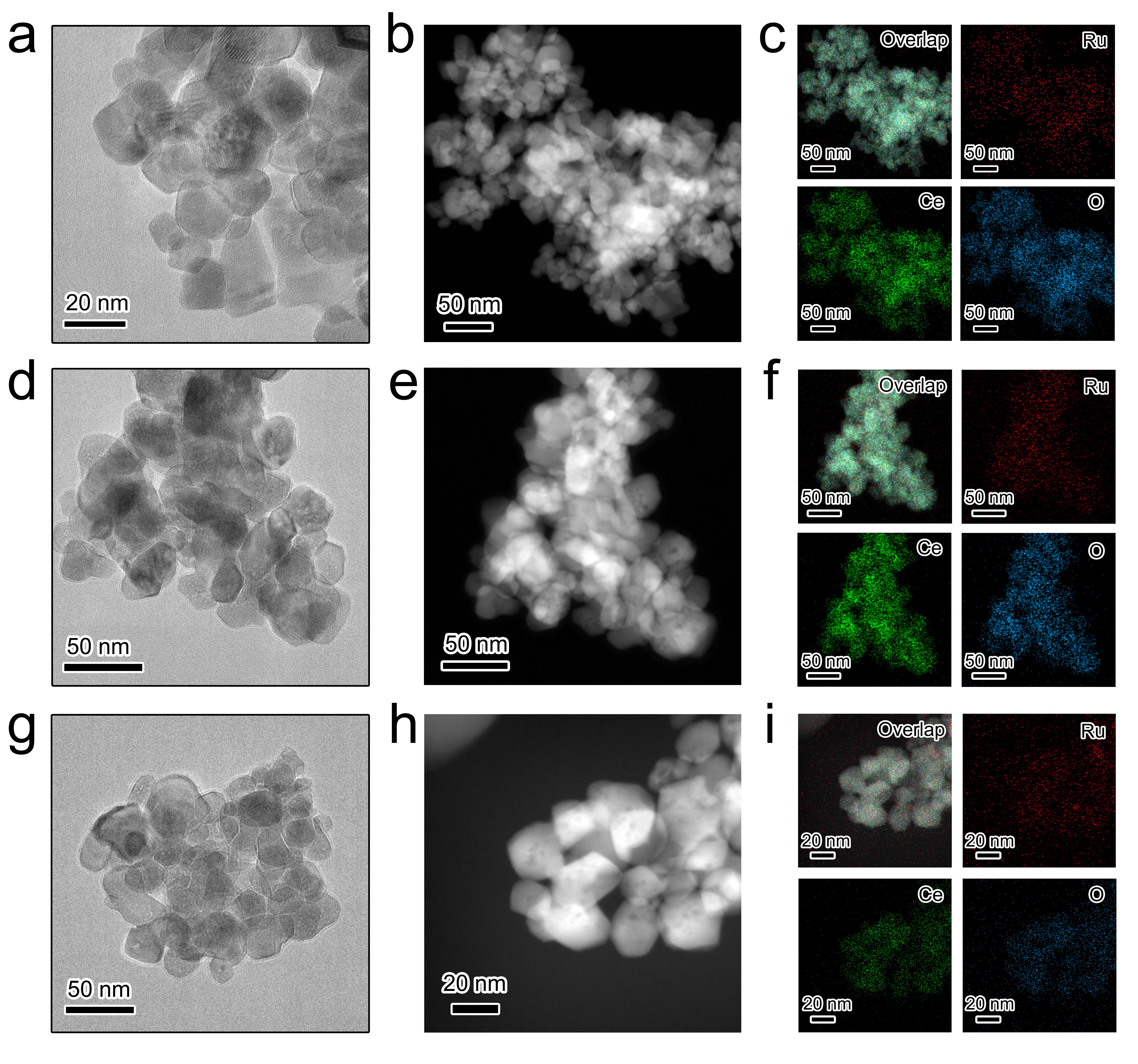


**Figure S2.** Structural analysis of 0.5Ru/CeO_2_, 2Ru/CeO_2_ and 5Ru/CeO_2_ catalysts. TEM, HAADF-STEM and corresponding elemental mapping images of (a−c) 0.5Ru/CeO_2_, (d−f) 2Ru/CeO_2_ and (g−i) 5Ru/CeO_2_ catalysts.





**Figure S3.** XRD patterns of various Ru catalysts.

**
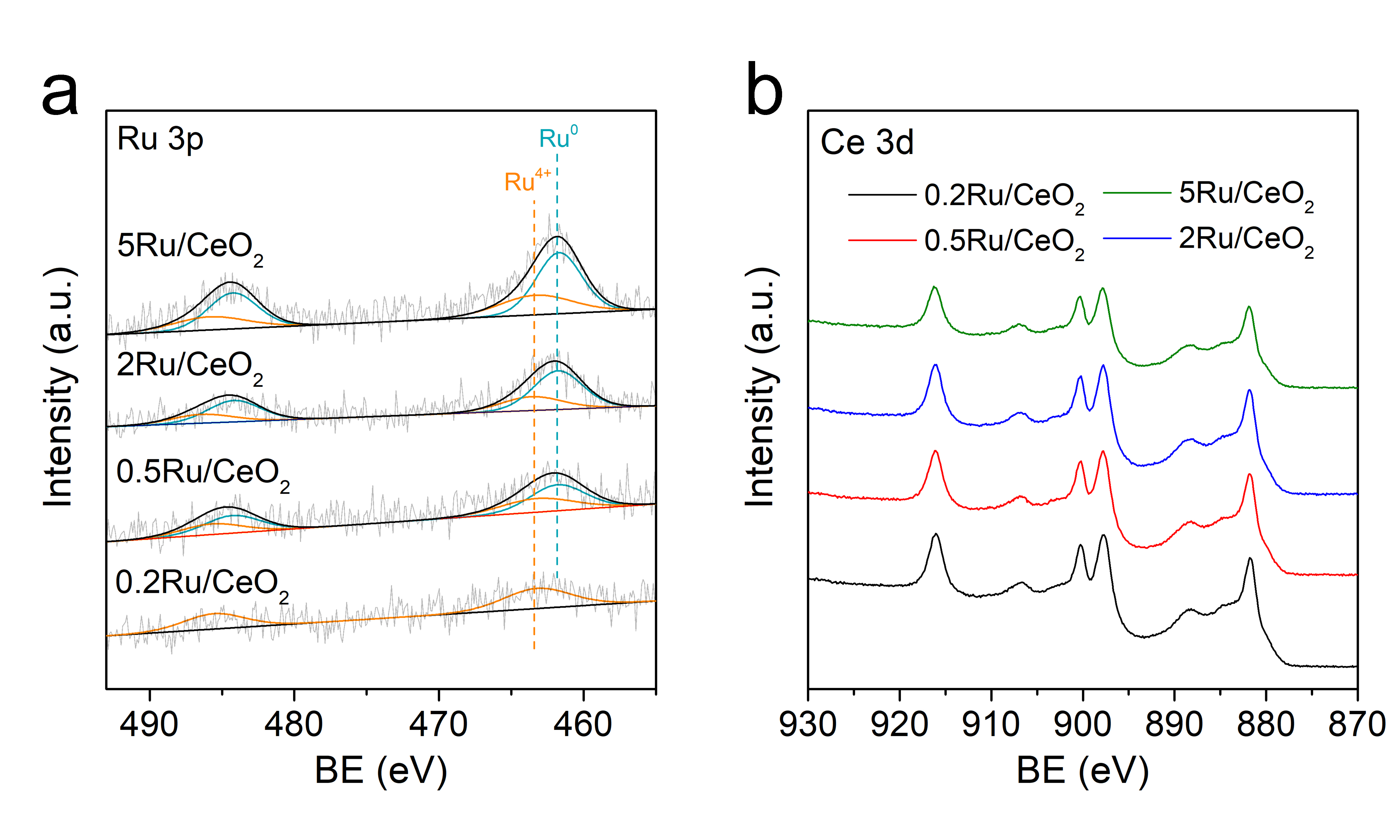
**

**Figure S4.** XPS spectra of various Ru catalysts at (a) Ru 3p and (b) Ce 3d orbitals.


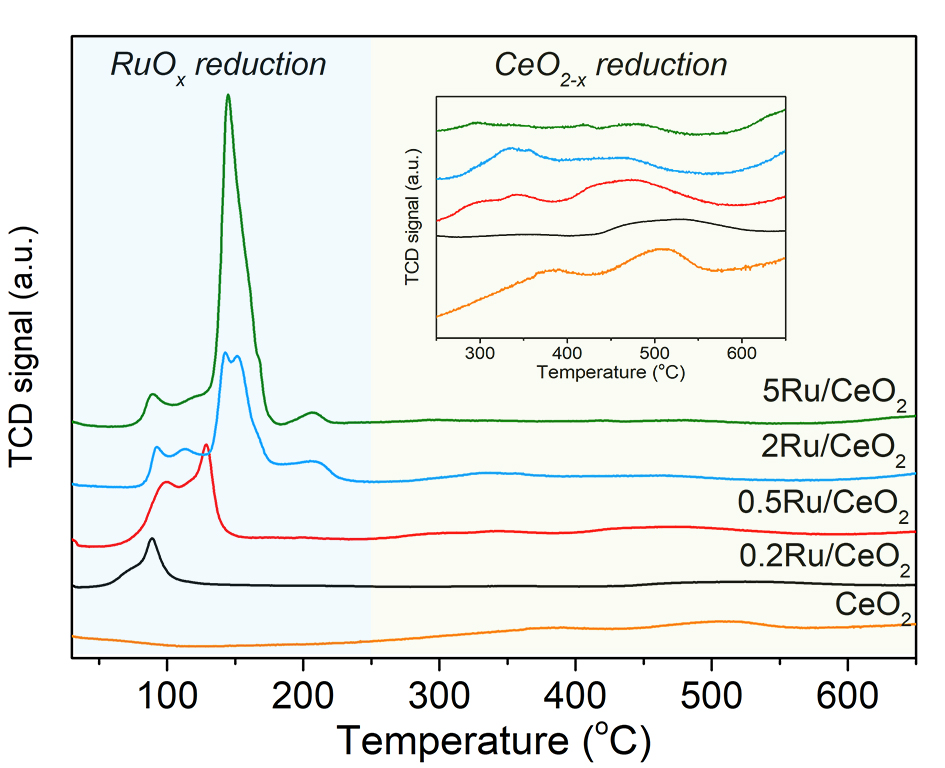


**Figure S5.** H_2_-TPR curve of various Ru catalysts and CeO_2_ support.

**
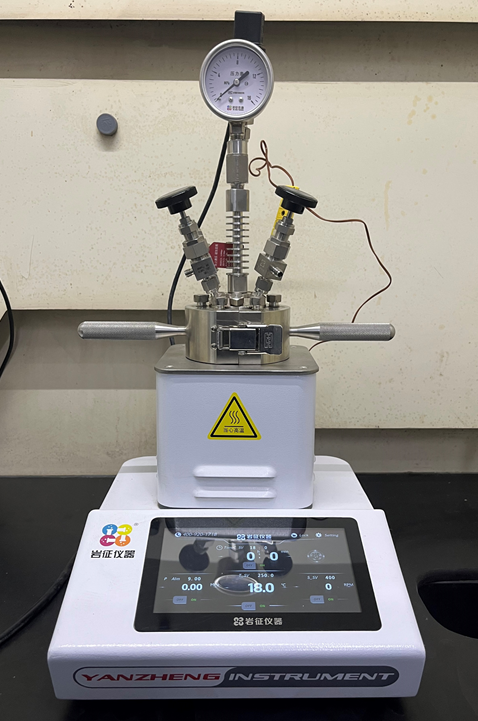
**

**Figure S6.** Picture of batch stainless-steel autoclave.

**
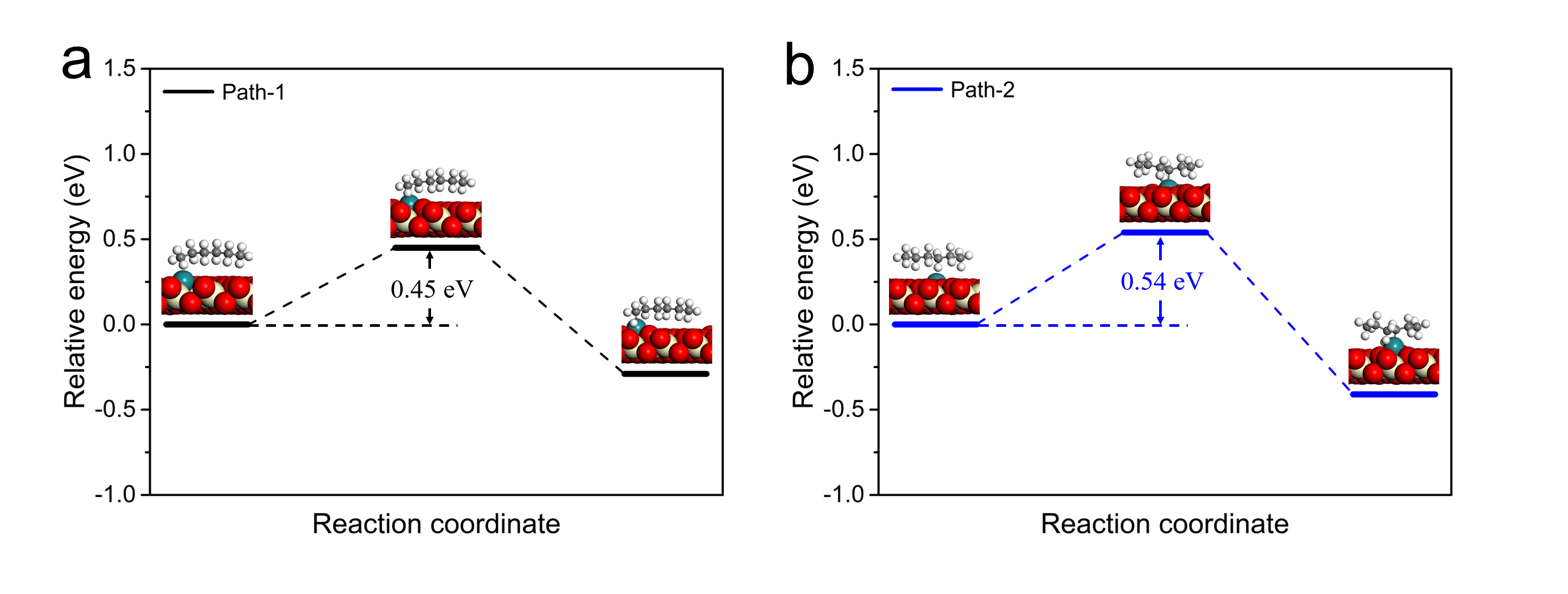
**

**Figure S7.** DFT calculations on C−H activation. (a, b) Reaction paths and activation barriers for cleavage of C−H bonds in *n*-hexane on single-atom Ru_1_/CeO_2_.


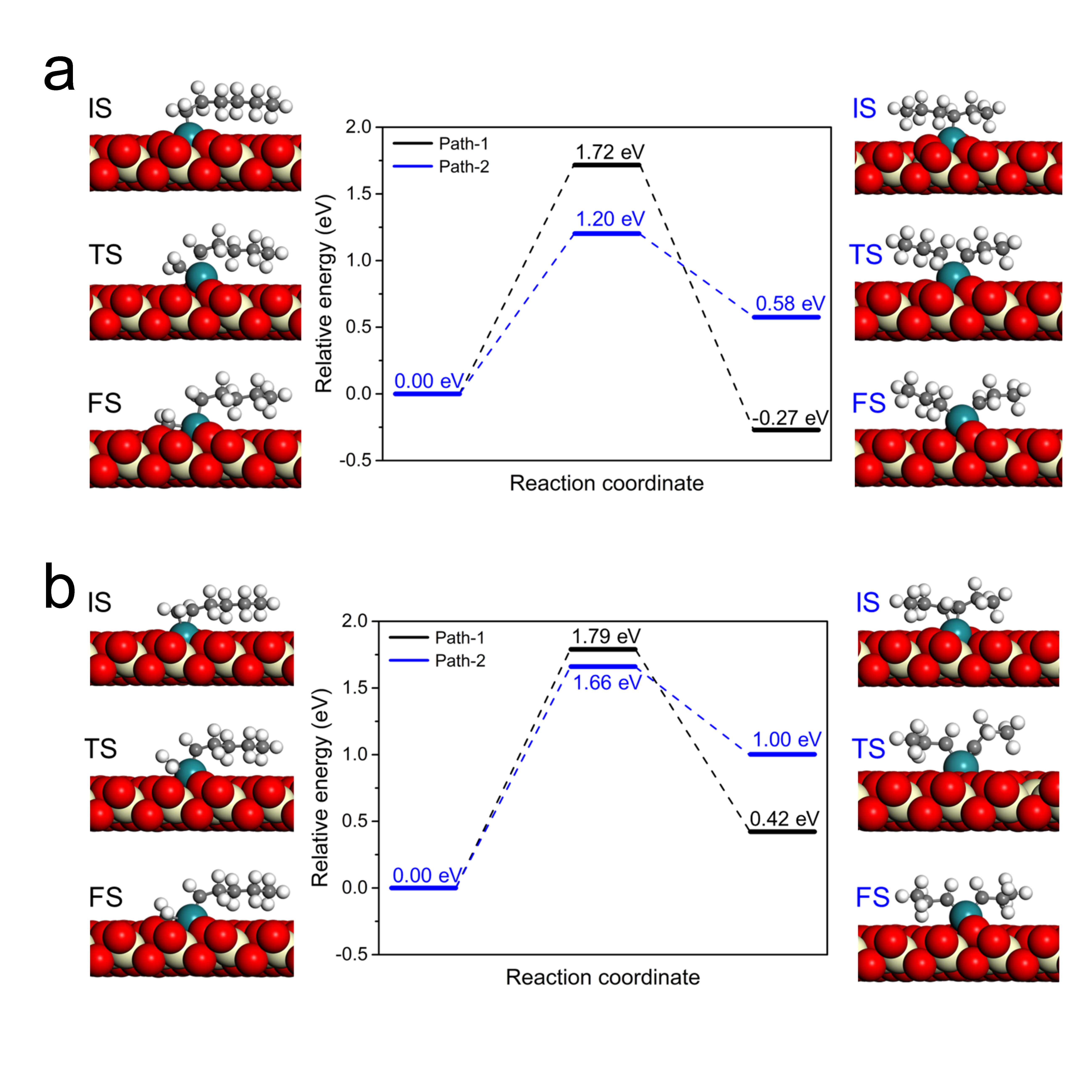


**Figure S8.** Theoretical calculations of C–C cleavage on Ru SAC. Reaction paths and activation barriers for cleavage of C–C bonds in *n*-hexane on Ru_1_/CeO_2_.

If the Ru atom is bonded with two contiguous carbon atoms, we found that the Ru_1_/CeO_2_ is also prone to activate the inner C3–C4 bond (1.66 eV) instead of the terminal C1–C2 bond (1.79 eV), but the activation barriers are all larger than those in Figure S8a, indicating a favorable geometric model of one carbon atom in hexene is bonded with a single Ru atom.


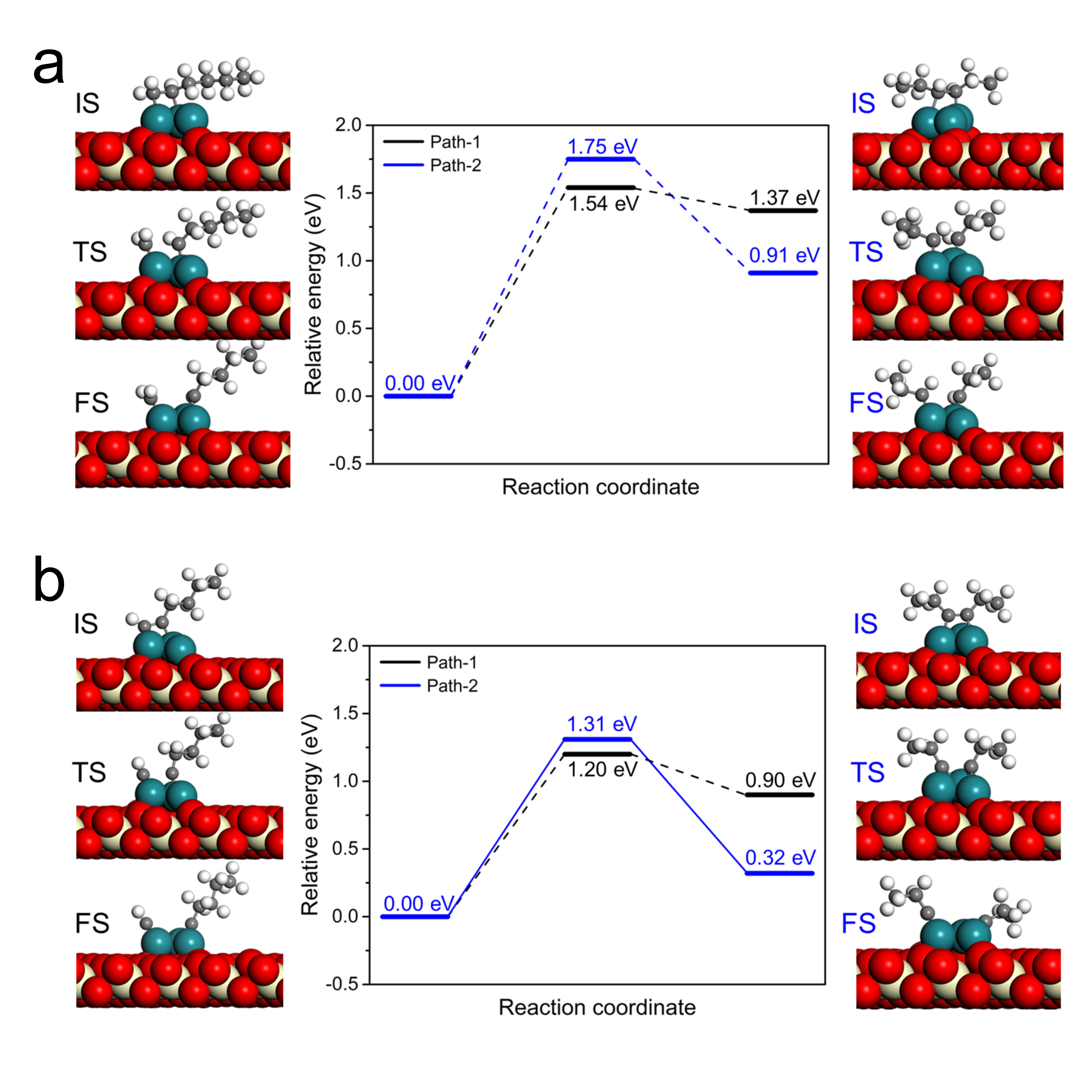


**Figure S9.** Theoretical calculations of C–C cleavage on Ru_3_/CeO_2_. Reaction paths and activation barriers for cleavage of C–C bonds in *n*-hexane on Ru_3_/CeO_2_.

By further dehydrogenation of hexene into hexyne, we have also evaluated the activation barriers for the C1–C2 bond and C3–C4 bond cleavage. To generate *CH and *CCH_2_CH_2_CH_2_CH_3_ intermediates by breaking the C1–C2 bond only needs to overcome a small barrier of 1.20 eV, in contrast to 1.31 eV by breaking the C3–C4 bond. The dissociated *CH intermediate bonds to a Ru atom and is possible to obtain CH_4_ as the final product. Therefore, the n-hexane on the Ru cluster is prone to dehydrogenated into 1-hexyne intermediate first, and then the terminal C–C bond will be cleaved to form CH_4_ as the product.


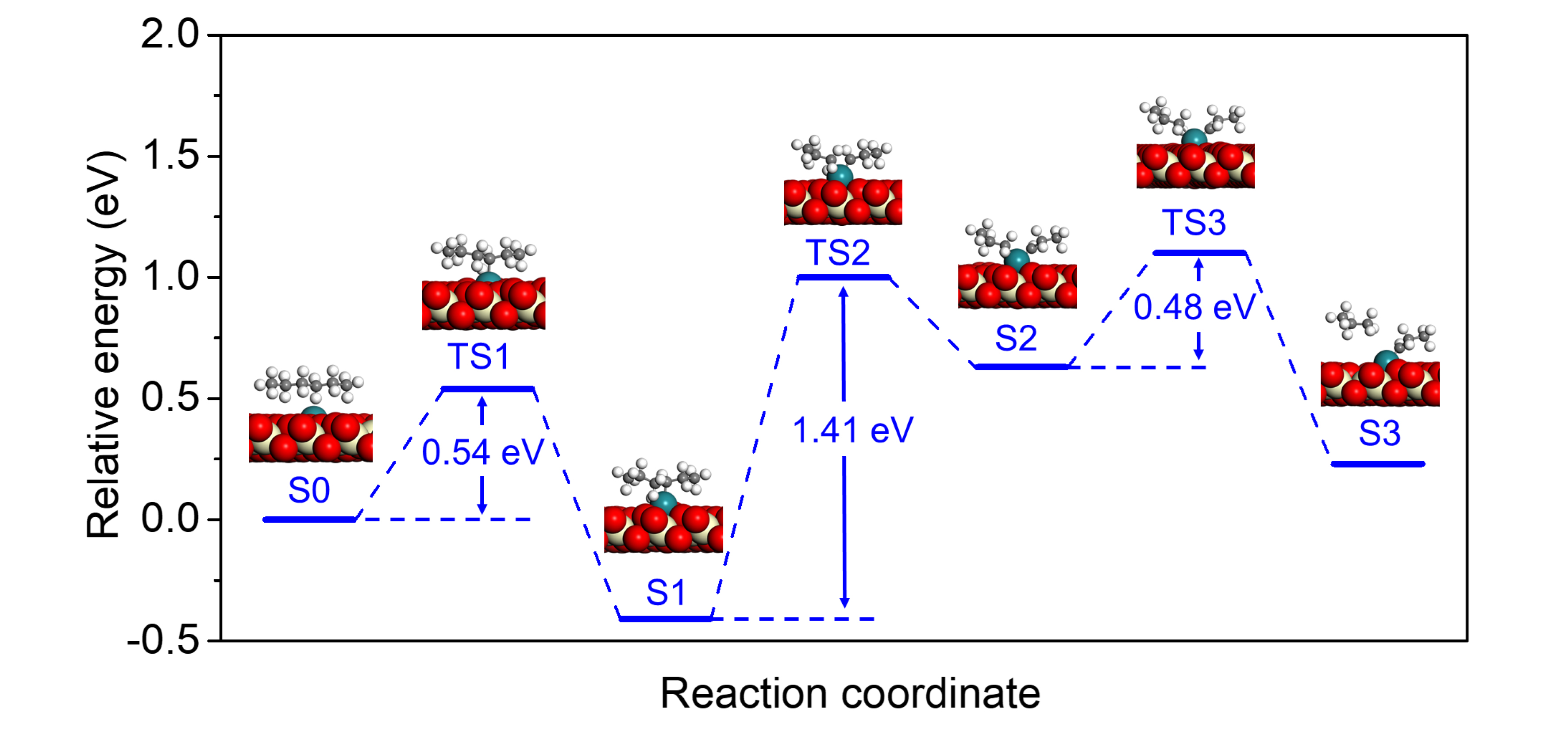


**Figure S10.** Catalytic reaction path and barriers of *n*-hexane dissociation on Ru SAC from DFT calculations.





**Figure S11.** Distribution of non-solid products on LDPE hydrogenolysis over Ru SAC from 2 to 24 h. Reaction conditions: T = 250 ^o^C, P_H2_ = 2 MPa, stirring rate = 400 rpm and m(Ru)/m(LDPE) = 1 mg/2000 mg.


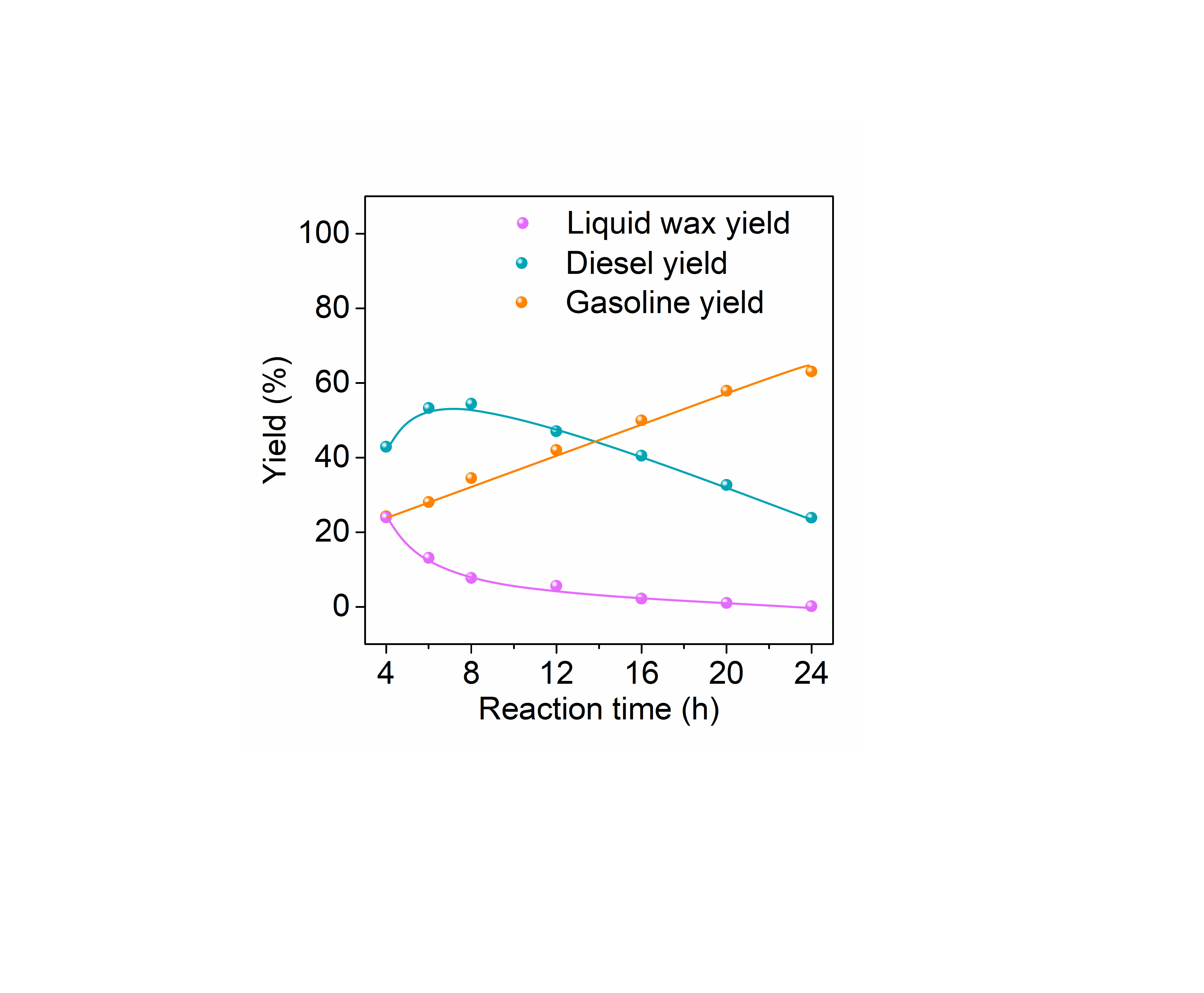


**Figure S12.** Time-dependent analysis. Product yields of gasoline (C5–C12), diesel (C13–C22) and liquid wax (C23–C35) over Ru SAC on LDPE hydrogenolysis for 4–24 h. Reaction conditions: T = 250 ^o^C, P_H2_ = 2 MPa, stirring rate = 400 rpm and m(Ru)/m(LDPE) = 1 mg/2000 mg.


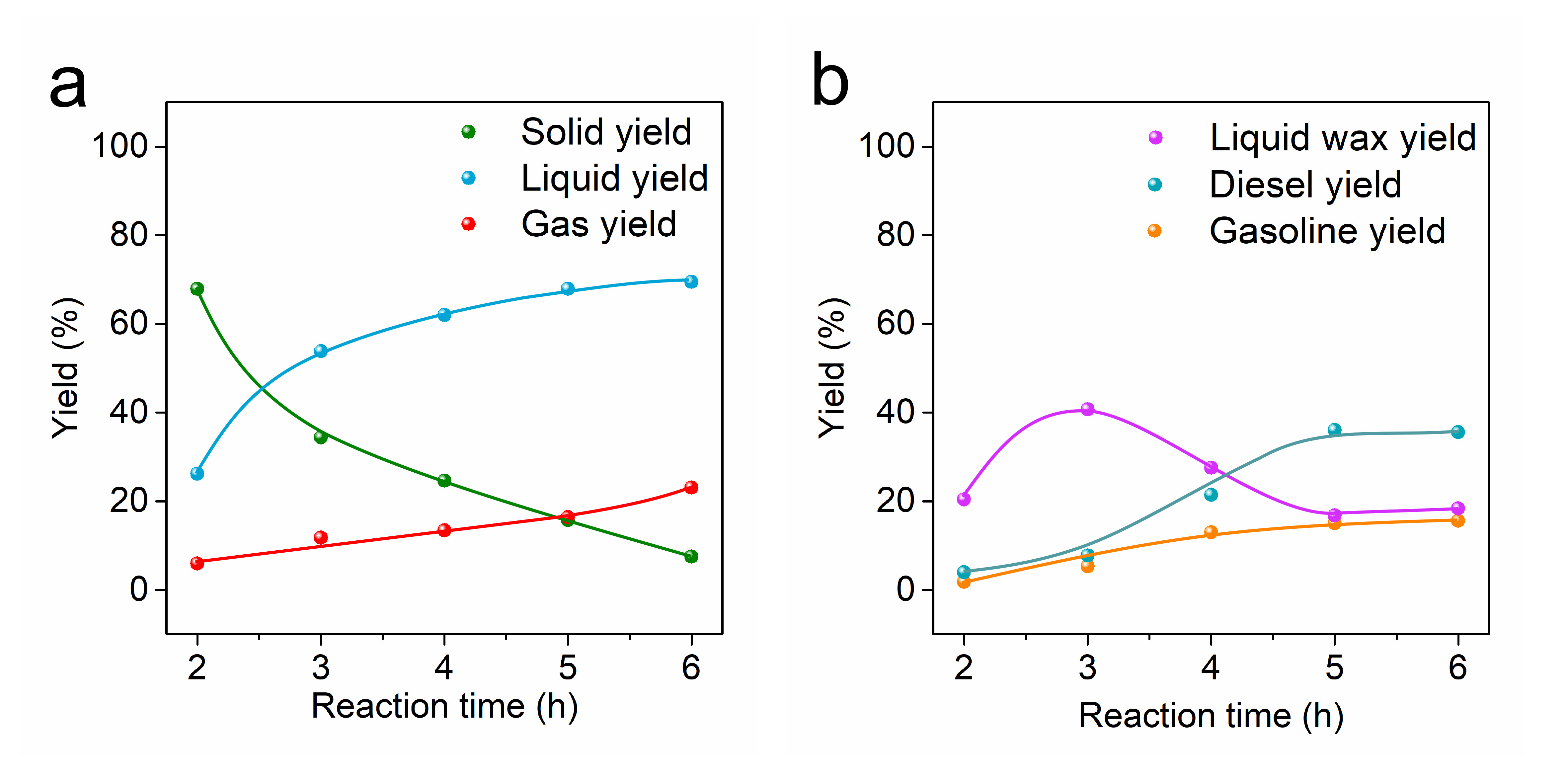


**Figure S13.** Time-dependent analysis of Ru non-SAC for 2−6 h. (a) Solid, liquid and gaseous yields. (b) Gasoline (C5−C12), diesel (C13−C22) and liquid wax (C22−C35) yields. Reaction conditions: T = 250 ^o^C, P_H2_ = 2 MPa, stirring rate = 400 rpm and m(Ru)/m(LDPE) = 1 mg/2000 mg.


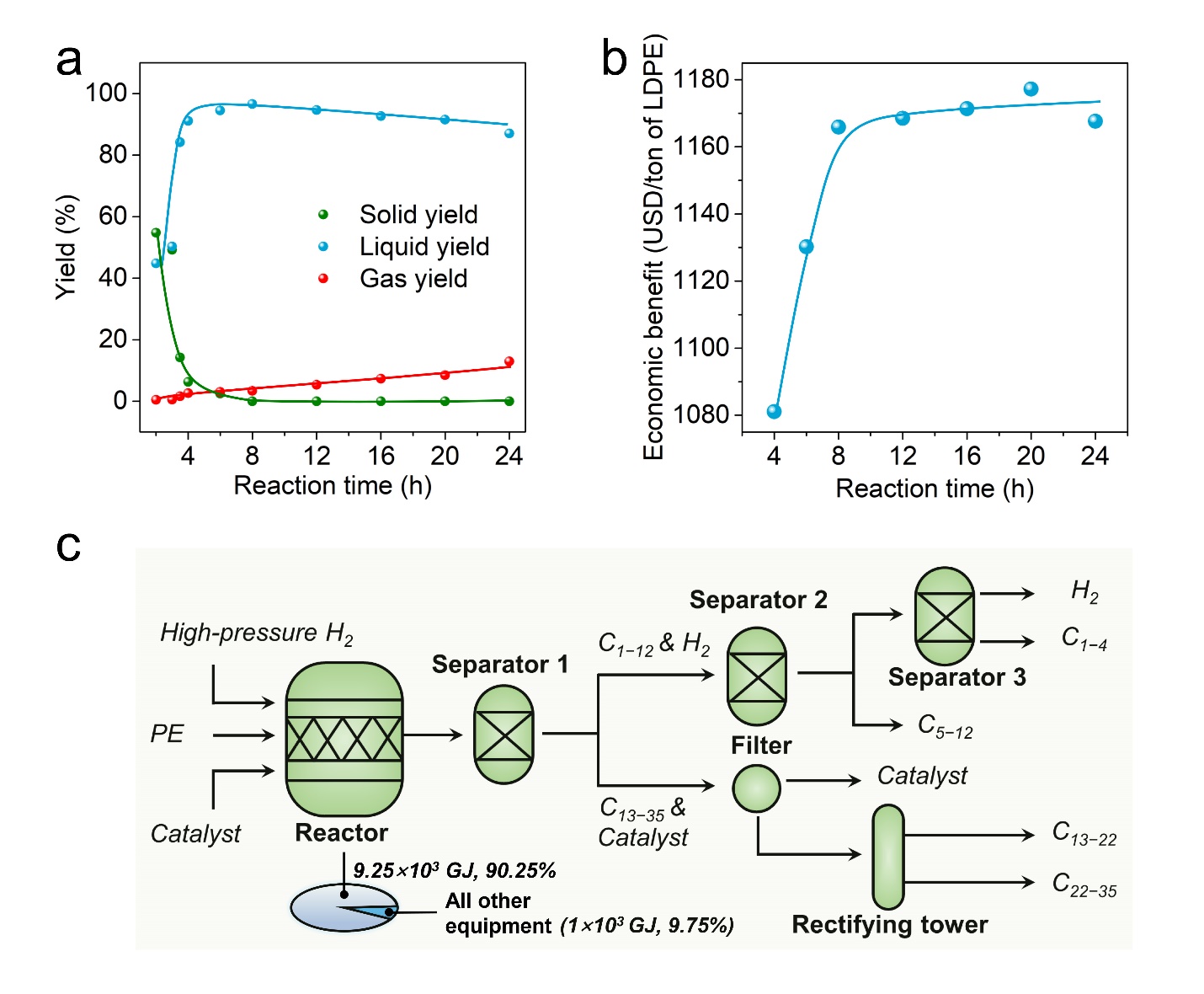


**Figure S14.** Schematic illustration of the industrial process of PE hydrogenolysis by Aspen Plus software. The blue pie chart represents the distribution of energy consumption for the reactor and other equipment.

**
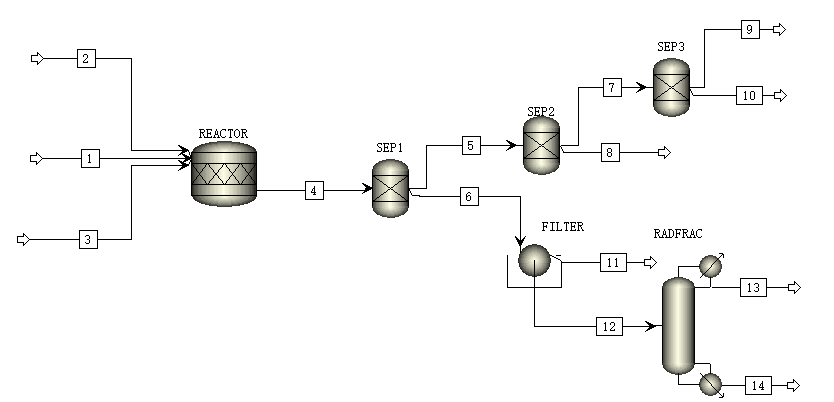
**

**Figure S15.** Detailed flow diagram of industrial PE hydrogenolysis based on the simulation through Aspen Plus software.


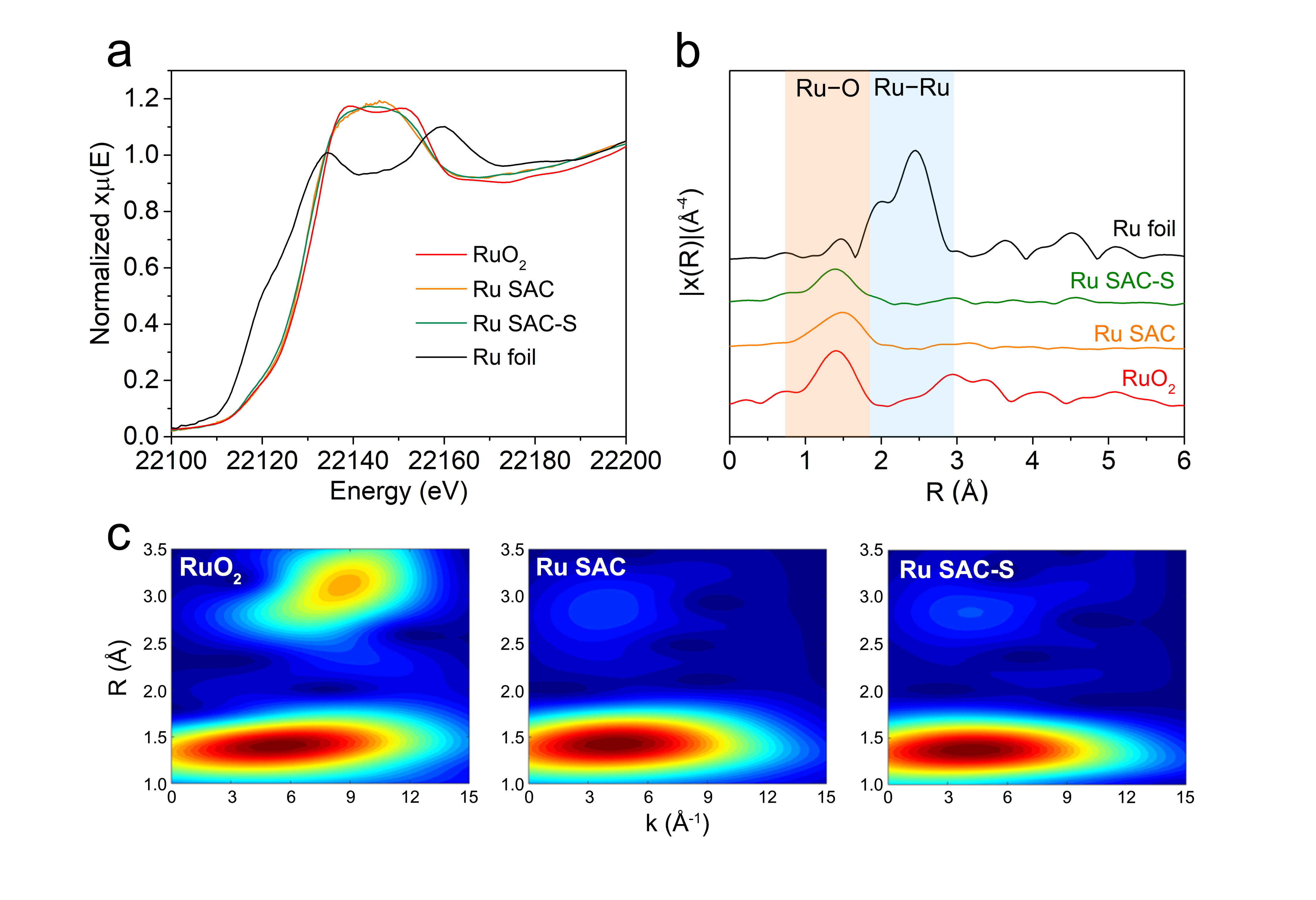


**Figure S16.** Electronic structure of Ru SAC-S. (a) XANES and (b) EXAFS spectra and (c) WT contour plots of fresh and spent Ru SACs as well as the references of Ru foil and RuO_2_.


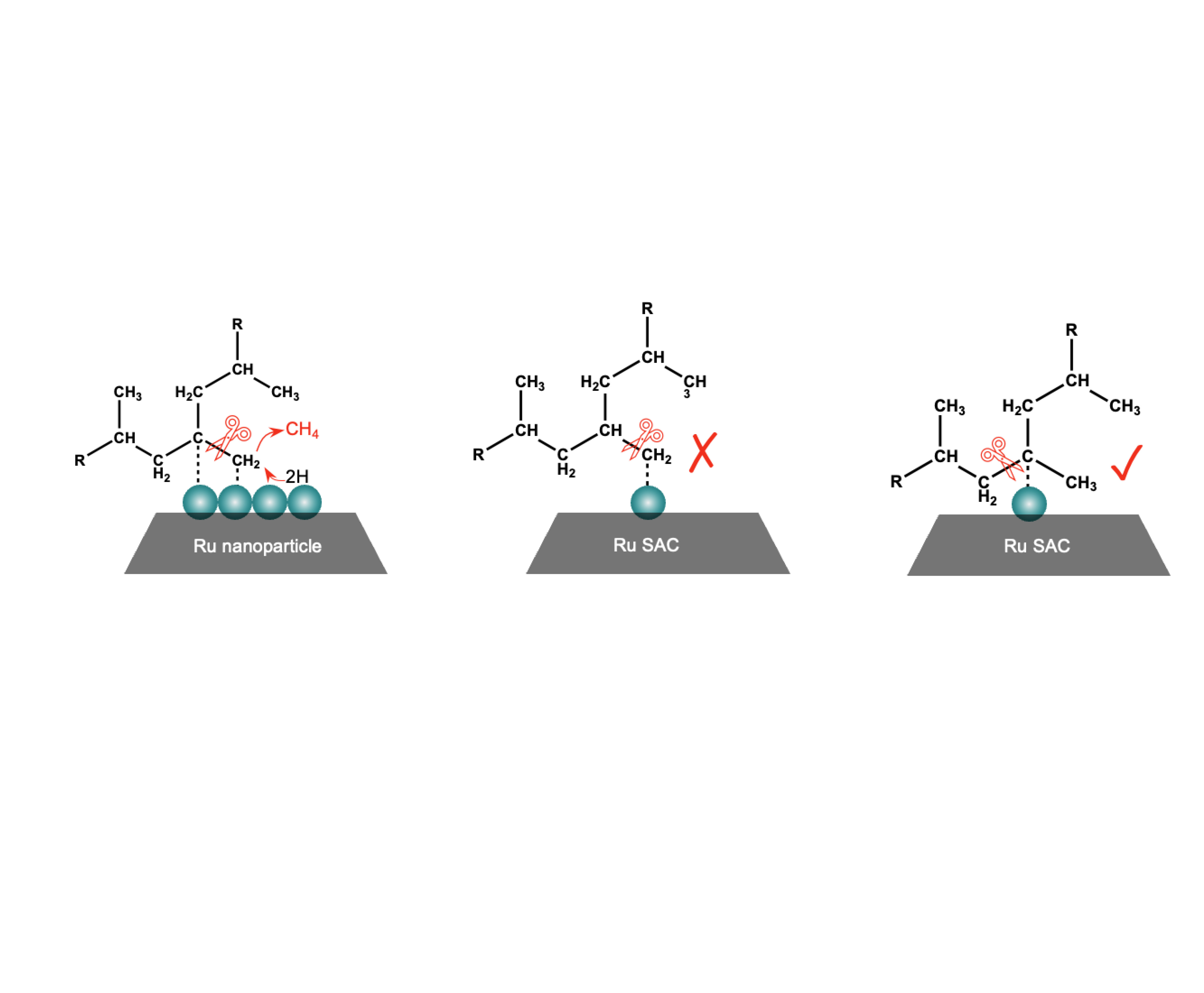


**Figure S17.** Plausible mechanism of C–C hydrogenolysis of polypropylene over Ru nanoparticle and Ru SAC.

**Table S1.** The elemental compositions of Ru/CeO_2_ catalysts measured by ICP-OES.

| Catalyst | Ru content (*wt*.%) |
| --- | --- |
| 0.2Ru/CeO_2_ | 0.18 |
| 0.5Ru/CeO_2_ | 0.53 |
| 2Ru/CeO_2_ | 2.12 |
| 5Ru/CeO_2_ | 4.89 |

**Table S2.** Fitting results of EXAFS spectra.

| Sample | Scatter path | CN | R(Å) | σ^2^(10^-3^ Å^2^) | ΔEo (eV) |
| --- | --- | --- | --- | --- | --- |
| Ru foil | Ru-Ru | 12 | 2.67 ± 0.04 | 2.84 ± 0.52 | 3.98 ± 0.79 |
| RuO_2_ | Ru-O | 6 | 1.97 ± 0.03 | 5.00 ± 0.21 | -3.71 ± 1.55 |
|  | Ru-Ru1 | 2 | 3.11 ± 0.01 | 3.54 ± 0.51 | -3.71 ± 1.55 |
|  | Ru-Ru2 | 8 | 3.55 ± 0.07 | 3.09 ± 0.95 | -3.71 ± 1.55 |
| 0.2Ru/CeO_2_ | Ru-O | 4.59 ± 1.17 | 2.02 ± 0.06 | 5.45 ± 3.98 | 2.12 ± 2.78 |
| 0.5Ru/CeO_2_ | Ru-O | 1.99 ± 0.56 | 2.01 ± 0.05 | 5.00 (fixed) | 5.95 ± 1.30 |
|  | Ru-Ru | 6.39 ± 1.03 | 2.67 ± 0.01 | 3.70 ± 0.96 | 5.95 ± 1.30 |
| 2Ru/CeO_2_ | Ru-O | 1.21 ± 0.33 | 1.94 ± 0.03 | 5.00 (fixed) | 4.35 ± 0.62 |
|  | Ru-Ru | 7.83 ± 0.26 | 2.67 ± 0.01 | 3.30 (fixed) | 4.35 ± 0.62 |
| 5Ru/CeO_2_ | Ru-O | 1.32 ± 0.79 | 1.99 ± 0.02 | 5.00 (fixed) | 4.53 ± 1.52 |
|  | Ru-Ru | 7.51 ± 1.45 | 2.67 ± 0.01 | 3.30 ± 1.17 | 4.53 ± 1.52 |
| 0.2Ru/CeO_2_-S | Ru-O | 4.42 ± 0.37 | 2.00 ± 0.04 | 5.45 (fixed) | 0.27 ± 3.46 |

**Table S3.** Summary and compassion of various Ru-based catalysts for polyolefin hydrogenolysis.

| **Catalyst** | **Plastic** | **T** | **t** | | **P_H2_** | | **P/C*^a^*** | **Gas yield** | **Liquid yield** | | **Solid yield** | | **Formation rate*^b^*** |
| --- | --- | --- | --- | --- | --- | --- | --- | --- | --- | --- | --- | --- | --- |
| Ru/C[8] | PE | 200 ^o^C | 16 h | | 20 bar | | 28 | 50% | 45% | | 5% | | 15.75 |
| Ru/TiO_2_[9] | i-PP | 250 ^o^C | 16 h | | 30 bar | | 20 | 28.2% | 65.6% | | 6.4% | | 13.90 |
| **Catalyst** | **Plastic** | **T** | **t** | | **P_H2_** | | **P/C*^a^*** | **C_1-4_ yield** | **C_5-21_ yield** | | **C_21+_ yield** | | **Formation rate*^b^*** |
| Ru/CeO_2_[10] | LDPE | 240 ^o^C | 8 h | | 3.5 MPa | | 34 | 10% | 84% | | 6.5% | | 76.50 |
| Ru/CeO_2_[10] | HDPE | 240 ^o^C | 10 h | | 3.5 MPa | | 34 | 13% | 83% | | 4.1% | | 59.16 |
| **Catalyst** | **Plastic** | **T** | **t** | | **P_H2_** | | **P/C*^a^*** | **Solvent** | **C_8-16_ yield** | | **C_17-22_ yield** | | **Formation rate*^b^*** |
| Ru/C[11] | HDPE | 220 ^o^C | 1 h | | 30 bar | | 2 | *n*-hexane | 60.8% | | 14.1% | | 29.96 |
| **Catalyst** | **Plastic** | **T** | **t** | **P_H2_** | | **P/C*^a^*** | | **Gas yield** | **CH_4_ yield** | **Liquid yield** | | **Formation rate*^b^*** | |
| Ru-15WZr[12] | LDPE | 250 ^o^C | 2 h | 50 bar | | 40 | | 11.8% | 8% | 61.2% | | 230.94 | |
| Ru/Zr[12] | LDPE | 250 ^o^C | 2 h | 50 bar | | 40 | | 21.1% | 16% | 53.9% | | 185.86 | |
| Ru-C[12] | LDPE | 250 ^o^C | 2 h | 50 bar | | 40 | | 26.9% | 20% | 39.1% | | 156.4 | |
| Ru-Ce[12] | LDPE | 250 ^o^C | 2 h | 50 bar | | 40 | | 17.5% | 14% | 33.5% | | 134.0 | |
| **Catalyst*^c^*** | **Plastic** | **T** | **t** | **P_H2_** | | **P/C*^a^*** | | **Gas yield** | **CH_4_ yield** | **Liquid yield** | | **Formation rate*^b^*** | |
| 0.2Ru/CeO_2_ | LDPE | 250 ^o^C | 6 h | 2 MPa | | 4 | | 3.05% | 2.23% | 94.5% | | 314.93 | |
| 0.5Ru/CeO_2_ | LDPE | 250 ^o^C | 6 h | 2 MPa | | 10 | | 16.8% | 15.75% | 76.63% | | 255.45 | |
| 2Ru/CeO_2_ | LDPE | 250 ^o^C | 6 h | 2 MPa | | 40 | | 23.06% | 21.1% | 69.45% | | 231.5 | |
| 5Ru/CeO_2_ | LDPE | 250 ^o^C | 6 h | 2 MPa | | 100 | | 62.5% | 58.6% | 4.46% | | 15.39 | |

*^a^* Mass ratio of plastic to catalyst.

*^b^* Formation rate (g_fuels_ g_Ru_^-1^ h^-1^) = m (liquid fuels)* m (Ru)^-1^* t^-1^

*^c^* Catalysts in our work (red)

**Table S4.** Summary of simulation parameters.

| **Component** | **Unit** | **1** | **2** | **3** | **4** | **5** | **6** | **7** | **8** | **9** | **10** | **11** | **12** | **13** | **14** |
| --- | --- | --- | --- | --- | --- | --- | --- | --- | --- | --- | --- | --- | --- | --- | --- |
| Stream | kg/h | 1000 | 50 | 250 | 1300 | 195.81 | 1104.2 | 42.81 | 153 | 40.4 | 2.45 | 250 | 854.2 | 609.55 | 244.64 |
| Temperature | C | 250 | 25 | 25 | 250 | 250 | 250 | 250 | 250 | 250 | 250 | 250 | 250 | 302.06 | 374.27 |
| Pressure | bar | 1 | 20 | 1 | 20 | 20 | 20 | 20 | 20 | 20 | 20 | 1 | 1 | 1 | 1 |
| PE | kg/h | 1000 | - | - | - | - | - | - | - | - | - | - | - | - | - |
| H_2_ | kg/h | - | 50 | - | 40.36 | 40.36 | - | 40.36 | - | 40.4 | - | - | - | - | - |
| Catalyst | kg/h | - | - | 250 | 250 | - | 250 | - | - | - | - | 250 | - | - | - |
| C1−C4 | kg/h | - | - | - | 2.45 | 2.45 | - | 2.45 | - | - | 2.45 | - | - | - | - |
| C5−C12 | kg/h | - | - | - | 153 | 153 | - | - | 153 | - | - | - | - | - | - |
| C13−C22 | kg/h | - | - | - | 611.94 | - | 611.9 | - | - | - | - | - | 611.9 | 606.2 | 5.74 |
| C23−C35 | kg/h | - | - | - | 242.25 | - | 242.3 | - | - | - | - | - | 242.3 | 3.35 | 238.9 |

**Table S5.** Energy consumption distribution of each component.

| **Component *^a^*** | **Energy consumption (GJ)** | **Proportion (%)** |
| --- | --- | --- |
| Reactor | 9.25 × 10^3^ | 90.25 |
| Separator 1 | -60.264 | -0.59 |
| Separator 2 | -140.4 | -1.37 |
| Rectifying tower | 1.2 × 10^3^ | 11.71 |
| Total | 1.02× 10^4^ | 100 |

*^a^* No energy consumption in separator 3 and filter.

**Table S6.** Comparison of energy consumption for complete conversion of PE into different products.

| **Product** | **Energy consumption (GJ)** |
| --- | --- |
| C5−C12 | 1.05× 10^4^ |
| C13−C22 | 1.16× 10^4^ |
| C23−C35 | 1.16× 10^4^ |

**References**

[1] G. Kresse, J. Furthmüller, "Efficient Iterative Schemes for Ab Initio Total-Energy Calculations Using a Plane-Wave Basis Set". *Physical Review B*, vol. 54, no. 16, pp. 11169-11186, 1996.

[2] P. E. Blochl, "Projector augmented-wave method". *Physical Review B*, vol. 50, no., pp. 17953-17979, 1994.

[3] J. P. Perdew, K. Burke, M. Ernzerhof, "Generalized Gradient Approximation Made Simple". *Physical Review Letters*, vol. 77, no. 18, pp. 3865-3868, 1998.

[4] S. Grimme, J. Antony, S. Ehrlich, H. Krieg, "A consistent and accurate ab initio parametrization of density functional dispersion correction (DFT-D) for the 94 elements H-Pu". *The Journal of Chemical Physics*, vol. 132, no. 15, pp. 154104, 2010.

[5] S. Dudarev, G. Botton, S. Savrasov, C. Humphreys, A. Sutton, "Electron-energy-loss spectra and the structural stability of nickel oxide: An LSDA+ U study". *Physical Review B*, vol. 57, no. 3, pp. 1505, 1998.

[6] T. Wu, T. Vegge, H. A. J. A. c. Hansen, "Improved electrocatalytic water splitting reaction on CeO_2_ (111) by strain engineering: a DFT+ U study". vol. 9, no. 6, pp. 4853-4861, 2019.

[7] G. Henkelman, B. P. Uberuaga, H. jónsson, "A climbing image nudged elastic band method for finding saddle points and minimum energy paths". *The Journal of Chemical Physics*, vol. 113, no. 22, pp. 9901-9904, 2000.

[8] J. E. Rorrer, G. T. Beckham, Y. Roman-Leshkov, "Conversion of polyolefin waste to liquid alkanes with Ru-based catalysts under mild conditions". *JACS Au*, vol. 1, no. 1, pp. 8-12, 2021.

[9] P. A. Kots, S. B. Liu, B. C. Vance *et al.*, "Polypropylene plastic waste conversion to lubricants over Ru/TiO2 catalysts". *ACS Catalysis*, vol. 11, no. 13, pp. 8104-8115, 2021.

[10] Y. Nakaji, M. Tamura, S. Miyaoka *et al.*, "Low-temperature catalytic upgrading of waste polyolefinic plastics into liquid fuels and waxes". *Applied Catalysis B: Environmental*, vol. 285, no., pp. 119805, 2021.

[11] C. Jia, S. Xie, W. Zhang *et al.*, "Deconstruction of high-density polyethylene into liquid hydrocarbon fuels and lubricants by hydrogenolysis over Ru catalyst". *Chem Catalysis*, vol. 1, no. 2, pp. 437-455, 2021.

[12] C. Wang, T. Xie, P. A. Kots *et al.*, "Polyethylene hydrogenolysis at mild conditions over ruthenium on tungstated zirconia". *JACS Au*, vol. 1, no. 9, pp. 1422-1434, 2021.
